# Molecular characterization and safety assessment of biofortified provitamin A rice

**DOI:** 10.1038/s41598-020-57669-5

**Published:** 2020-01-28

**Authors:** Norman Oliva, Maria Florida Cueto-Reaño, Kurniawan R. Trijatmiko, Mercy Samia, Ralf Welsch, Patrick Schaub, Peter Beyer, Donald Mackenzie, Raul Boncodin, Russell Reinke, Inez Slamet-Loedin, B. P. Mallikarjuna Swamy

**Affiliations:** 1 Strategic Innovation Platform, International Rice Research Institute (IRRI), Metro Manila, Philippines; 2 Faculty of Biology, Cell Biology, University of Freiburg, Freiburg, Germany; 3 Institute for International Crop Improvement, Donald Danforth Plant Science Center, Saint Louis, Missouri USA

**Keywords:** Agricultural genetics, Agricultural genetics, Agricultural genetics

## Abstract

Part of the studies involved in safety assessment of genetically engineered crops includes characterizing the organization, integrity, and stability of the inserted DNA and evaluating the potential allergenicity and toxicity of newly-expressed proteins. Molecular characterization of the introduced DNA in provitamin A biofortified rice event GR2E confirmed insertion of a single copy of the transfer-DNA in the genome and its inheritance as a single locus. Nucleotide sequencing of the inserted DNA confirmed it was introduced without modifications. The phytoene synthase, and carotene desaturase proteins did not display sequence similarity with allergens or toxins. Both proteins were rapidly digested in simulated gastric fluid and their enzymatic activity was inhibited upon heat treatment. Acute oral toxicity testing of the protein in mice demonstrated lack of adverse effects. These evidences substantiated the lack of any identifiable hazards for both proteins and in combination with other existing comparative analyses provided assurance that food derived from this rice is safe. This conclusion is in line with those of the regulatory agencies of US Food and Drug Administration, Health Canada and Food Standard Australia and New Zealand.

## Introduction

Vitamin A deficiency (VAD) persists as a major public health nutrition problem, especially in sub-Saharan Africa and South Asia. Severe deficiency can lead to disorders such as xerophthalmia, the leading cause of preventable childhood blindness, anemia, and weakened host resistance to infection, which can increase the severity of infectious diseases and risk of death^1,2^. VAD increases vulnerability to a range of illnesses including diarrhea, measles, and respiratory infections, which are the leading causes of mortality among children in low and middle income countries, where risk of infection and risk of mortality can be compounded by coexisting under-nutrition. The prevalence and severity of xerophthalmia, anemia and the less-measurable “vicious cycle” between VAD and infection in vulnerable groups, notably young children and pregnant or lactating mothers, represent the most compelling consequences of VAD and underscore its significance as a public health problem around the world. In Bangladesh, the prevalence of VAD among preschool and school age children is a serious public health problem, with respective rates found to be 20.5 percent and 20.9 percent, respectively^3^.

In Southeast Asian countries, the average per capita milled rice consumption is 130 kg/year or 360 g/day, providing nearly two-thirds of the necessary caloric intake. The high consumption of rice may reflect a lack of dietary diversity, which, when combined with the poor micronutrient content of polished rice in general and lack of any provitamin A, specifically, is a risk factor for VAD^4^. In addition to existing efforts, a complementary intervention for reducing VAD in the highest-risk countries is the biofortification of staple food crops with provitamin A. This approach has been successfully used with crops such as maize, cassava, and sweet potato where existing germplasm with elevated levels of provitamin A expression has been introduced into breeding programs^5,6^.

Golden Rice event 2E (GR2E) was developed through the use of recombinant-DNA techniques to express elevated levels of provitamin A (mainly ß-carotene) in the endosperm, which is converted in the body to vitamin A. The use of recombinant DNA technologies was required because provitamin A formation in the grain endosperm was not found in rice germplasm collections. GR2E rice provides a complementary effort to reduce VAD by providing ready access to a portion of the estimated average requirement for vitamin A deficient population whose diet is primarily rice-based.

The carotenoid biosynthetic pathway in immature rice endosperm functions up to the synthesis of geranylgeranyl diphosphate. While this compound is not solely devoted to carotenogenesis, it can be used as a substrate to produce the phytoene by a phytoene synthase heterologously expressed in the rice endosperm^7^. Completion of the pathway leading to the synthesis of ß-carotene via expression of the carotene desaturase I (*crtI*) gene from *Pantoea ananatis* (formerly *Erwinia uredovora*) that is fused in-frame at the 5′ terminus with the pea (*Pisum sativum*) RUBISCO SSU transit peptide encoding sequence (*SSU-crtI*), was described by Ye *et al*.^8^. Paine *et al*.^9^ described improved constructs incorporating the *Zea mays* phytoene synthase gene (*Zmpsy1*) and phosphomannose isomerase (*pmi)* as selection marker for transformants, which produced elevated total carotenoid levels of up to 30 μg/g in the endosperm, of which *ca*. 80 percent were mixed isomers of ß-carotene. One of the resulting *Agrobacterium tumafaciens-* mediated transformants was GR2E using plasmid pSYN12424 containing the *SSU-crtI* (from *Pisum sativum* and *Pantoea ananatis*), *Zmpsy1* and *pmi* (from *E.coli*).

Completion of the food safety assessment is a necessary prerequisite to the conduct of bioefficacy studies with GR2E, which are an important component in demonstrating the value of GR2E rice in mitigating VAD. The totality of the assessment that provides assurance that GR2E is as safe as conventional rice involves characterization of introduced genes and proteins, which is discussed in this paper and also additionally, comparative analysis of the composition of rice used for food and feed^10^. Molecular characterization of a new transgenic event is important in providing an understanding of the nature of the genetic modification; including any genetic material introduced into the host genome, and provides context for the safety assessment^11^. Typically, the molecular characterization addresses three main aspects: a) the transformation method together with a detailed description of the DNA sequences introduced into the host genome; b) characterization of the inserted DNA, including any rearrangements that may have occurred as a consequence of the transformation; and c) the genetic stability of the inserted DNA and any expressed traits. The molecular characterization of GR2E rice included Southern blot analyses to confirm the number of sites of insertion of the transfer-DNA (T-DNA), the absence of plasmid backbone sequences, and the multi-generational stability of the inserted DNA. Nucleotide sequencing of the entire T-DNA insert, including a portion of the 5′ and 3′flanking host genomic region was carried out in order to demonstrate overall integrity of the insert, contiguity of the functional elements, and to detect any individual base-pair changes. Bioinformatic analyses were used to investigate the possibility of the unintended creation of any novel open reading frames (ORFs) spanning the inserted and genomic DNA junctions, potentially encoding allergens or toxins. Finally, segregation analyses served to demonstrate Mendelian inheritance of the introduced genes and traits.

Assessing the safety of newly expressed proteins produced in the edible portions of a genetically engineered food crop is an integral component of the overall safety assessment. There is currently no single criterion that is sufficiently predictive of potential toxicity or allergenicity. Therefore, a “weight-of-evidence” approach is recommended for hazard identification that considers the history of safe use, amino acid sequence similarity to known toxins or allergens, function or mode of action, digestibility under standardized *in vitro* conditions, stability to heat or processing, and expression levels and potential dietary exposure^12,13^. Depending on the results from early tier evaluation, additional characterization may include appropriate oral toxicity studies or other hypothesis-based toxicology studies. This approach was followed in assessing the safety of the phytoene synthase (*Zm*PSY1) and carotene desaturase (CRTI) proteins expressed in GR2E rice, and it is described in the following sections.

Based on the presence of PMI in a wide range of food and feedstuffs derived from genetically engineered maize lines, and on the extensive history of prior regulatory reviews^14^ in some countries, additional characterization of the protein was unnecessary. Previously submitted safety studies^15–19^ reviewed in the context of other genetically engineered plant events are directly applicable to the safety assessment of PMI protein expressed in GR2E rice.

The data on the molecular characterization and safety assessment of the proteins in GR2E was part of the regulatory dossier that is intended to meet the regulatory requirements in target countries such as Philippines, Bangladesh, and Indonesia. Other countries may have other requirements.

## Results

### Southern blot characterization of GR2E rice

*Hin*dIII and *Sph*I both with unique restriction sites within the T-DNA (Supplementary Fig. 1, adapted from submitted GR2E-FFP (Food and Feed or for Processing) study reports) were used for determining the copy number of the introduced DNA within the GR2E genome. Hybridization of *Hin*dIII-digested genomic DNA from homozygous plants of GR2E in four genetic backgrounds (Kaybonnet, PSBRc82, BRRI *dhan*29, and IR64) with the *Zmpsy1* or *pmi* probes resulted in the detection of a single fragment of *ca*. 7900 bp, and hybridization with the probe specific for *SSU-crtI* yielded a single *ca*. 7200 bp fragment (Supplementary Table 2, Supplementary Fig. 2, lanes 16–19, adapted from GR2E-FFP submitted study reports). Hybridization of *Sph*I-digested GR2E genomic DNA with the *Zmpsy1* or *SSU-crtI* probes gave a single fragment of *ca*. 6900 bp, and upon hybridization with the *pmi* probe a single fragment of *ca*. 5500 bp was observed (Supplementary Fig. 2, lanes 6–9, adapted from GR2E-FFP submitted study reports; Supplementary Table 2). Weak hybridization between the *Zmpsy1* probe and sequences derived from the endogenous rice *psy1* gene was detected for restriction enzyme digests of control Kaybonnet and GR2E DNA samples (Supplementary Fig. 2, panel A, adapted from GR2E-FFP submitted study reports). Hybridizing fragments of *ca*. 5600 bp and *ca*. 4900 bp were detected in Southern blots of *Sph*I and *Hin*dIII-digested DNA samples, respectively. This was not an unexpected finding considering the high degree of sequence identity, ca. 83 percent, shared between the *Zmpsy1* and *Oryza sativa psy* genes.

Southern blot analyses of *Asc*I + *Xma*I-digested GR2E rice DNA were used to investigate the integrity of the T-DNA insert containing the *Zmpsy1*, *SSU-crtI*, and *pmi* gene cassettes. The T-DNA contains a single *Asc*I restriction site located at position 199 and a single *Xma*I site at position 8946 (Supplementary Fig. 1, adapted from GR2E-FFP submitted study reports). Insertion of an intact copy of the T-DNA should thus result in the detection of an 8747 bp *Asc*I + *Xma*I fragment with the *Zmpsy1*, *SSU-crtI*, and *pmi* probes. The results of Southern analyses (Supplementary Fig. 2, lanes 11–14, adapted from GR2E-FFP submitted study reports) demonstrate that the correct size fragment was detected with all of the hybridization probes (Supplementary Table 2).

Hybridizing fragments were not detected when backbone probes were tested against samples of *Asc*I + *Xma*I-digested GR2E genomic DNA (Supplementary Fig. 3, lanes 6–8, panels A and B, adapted from GR2E-FFP submitted study reports), confirming the absence of plasmid backbone sequences. Positive control samples of wild-type Kaybonnet genomic DNA spiked with pSYN12424 plasmid DNA did result in detection of the expected-size 4349 bp fragment using backbone probe 5 (Supplementary Fig. 3, panel B, lane 3, adapted from GR2E-FFP submitted study reports) and two fragments of 1243 bp and 4349 bp, respectively, using the mixture of backbone probes 1–4 (Supplementary Fig. 3, panel A, lane 3, adapted from GR2E-FFP submitted study reports).

Thus, multiple Southern hybridization analyses clearly demonstrate the insertion of the T-DNA into a single site and the absence of sequences derived from the plasmid backbone.

### Stability of the introduced trait across multiple generations

**Figure 1:**
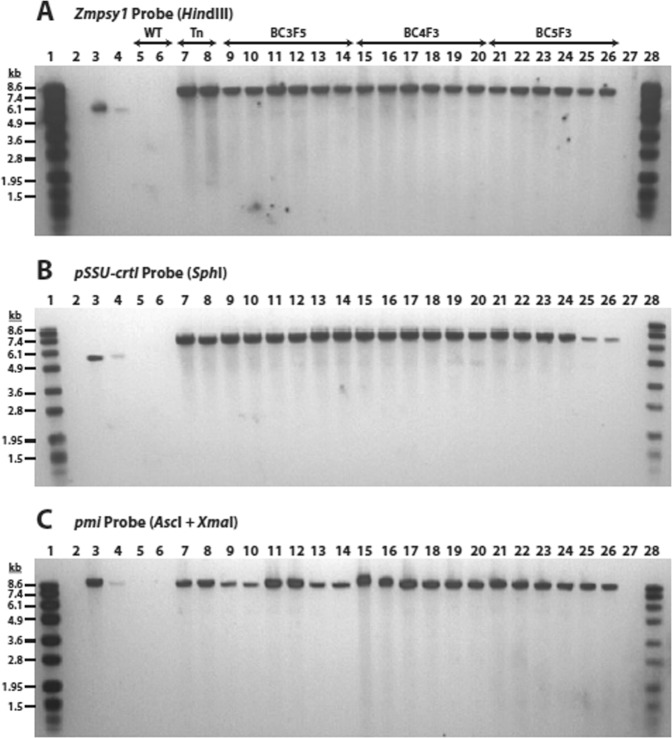
Samples of DNA from individual plants of event GR2E in Kaybonnet (Tn; lanes 7–8, as replicates), BRRI dhan 29 (BC_3_F_5_, lanes 9–10; BC_4_F_3_, lanes 15–16; and BC_5_F_3_, lanes 21–22), IR64 (BC_3_F_5_, lanes 11–12; BC_4_F_3_, lanes 17–18; and BC_5_F_3_, lanes 23–24), and PSB Rc82 (BC_3_F_5_, lanes 13–14; BC_4_F_3_, lanes 19–20; and BC_5_F_3_, lanes 25–26) germplasm backgrounds and negative control DNA from Kaybonnet rice (lanes 5–6) were subjected to Southern blot analysis. For this, 5 μg genomic DNA was digested with HindIII (panel A), SphI (panel B), or AscI plus XmaI (panel C) followed by agarose gel electrophoresis and transfer onto nylon membrane. Positive control samples consisted of negative control Kaybonnet rice containing either one (lane 3) or 0.2 (lane 4) copy equivalents of pSYN12424 plasmid DNA digested with SphI (panels A and B) or AscI + XmaI (panel C), Blots were hybridized with DIG-labelled probes for Zmpsy1 (panel A), pSSU-crtI (panel B), or pmi (panel C). Following washing, hybridized probes and DIG-labelled molecular weight markers VII (lanes 1 and 28) were visualized using a chemiluminescent detection. Lanes 2 and 27 were blank on all gels (adapted from GR2E-FFP submitted study reports).

The stability of the inserted DNA across multiple generations was assessed by Southern blot analyses of genomic DNA samples prepared from a selfed generation of GR2E in Kaybonnet background (T_n_) and three back-cross generations of GR2E (BC_3_F_5_, BC_4_F_3_, and BC_5_F_3_) for each recurrent parents (BRRI *dhan* 29, IR64 and PSBRc82). Digestions with *Hin*dIII, *Sph*I and *Asc*I + *Xma*I were separated by gel electrophoresis and blots were probed with probes specific for *Zmpsy1*, *SSU-crtI*, or *pmi* genes, respectively (Fig. 1, adapted from GR2E-FFP submitted study reports). Single hybridizing fragments of ~7900 bp, ~6900 bp, or 8747 bp were detected using the *Zmpsy1, SSU-crtI*, or *pmi* probes, respectively, in corresponding blots of *Hin*dIII, *Sph*I, or *Asc*I + *Xma*I digests of genomic DNA from each generation of GR2E rice (Supplementary Table 3).

Concentrations of total carotenoids were determined in grain samples collected from GR2E plants in Kaybonnet germplasm, the BC_3_F_5_ generations in PSBRc82 and IR64 backgrounds, and the BC_4_F_3_ and BC_5_F_3_ generations in PSBRc82, IR64, and BRRI *dhan* 29 germplasm backgrounds (Table 1). Carotenoid accumulation in the endosperm was positively correlated with the presence of the T-DNA insert as previously established by Southern blot characterization of the same generations and germplasm backgrounds of GR2E rice. Some variation in the concentrations of total carotenoids was observed depending on the germplasm background, with Kaybonnet and BRRI *dhan* 29 GR2E attaining the highest levels.

**Table 1:** Concentrations of total carotenoids in different generation and germplasm backgrounds of GR2E rice.

Breeding Generation	Total Carotenoids (µg/g FWT)^†^			
	Kaybonnet	PSB Rc82	IR64	BRRI *dhan* 29
T(n)	30.50 ± 2.49	—	—	—
BC_3_F_5_	—	12.84 ± 3.32	20.11 ± 8.71	ND^a^
BC_4_F_3_	—	9.09 ± 2.26	19.40 ± 2.00	24.50 ± 2.85
BC_5_F_3_	—	14.12 ± 0.11	13.23 ± 1.16	29.33 ± 3.14

^†^Values shown are mean values ± SD (standard deviation) determined spectrophotometrically for total carotenoids in grain samples after one day of storage at 16 °C. All concentrations are on a fresh weight (FWT) basis, not corrected for moisture content.

^a^ND = Not determined. Due to poor quality of remnant seed from the BC_3_F_5_ generation of BRRI *dhan* 29 containing GR2E, there was no seed germination and plants could not be produced for grain sampling.

### Mendelian inheritance of the inserted DNA

The inheritance pattern of the T-DNA insert within GR2E rice was investigated using a polymerase chain reaction (PCR)-based zygosity test. Segregation of the insert within three segregating generations (BC_4_F_2_, BC_5_F_1_, and BC_5_F_2_) in each of three genetic backgrounds was determined. Chi-square analysis resulted in no statistically significant differences between the observed and expected segregation ratios for the three segregating generations of GR2E in PSBRc82, BRRI *dhan*29, and IR64 genetic backgrounds (Supplementary Table 5).

### Nucleotide sequence analysis of the inserted DNA and flanking regions

The nucleotide sequence of the plasmid T-DNA, together with preliminary sequence information from the 5′ and 3′ flanking genomic DNA, was used to design seven sets of oligonucleotide primers to amplify the insert and flanking regions from GR2E genomic DNA as seven individual overlapping fragments (Supplementary Fig. 4, adapted from GR2E-FFP submitted study reports).

In total, 12,772 bp of GR2E genomic sequence was determined, comprising 1,988 bp of the 5′ genomic border sequence, 1,788 bp of the 3′ genomic border sequence, and 8,996 bp of the inserted T-DNA (Supplementary Fig. 4, adapted from GR2E-FFP submitted study reports). The T-DNA in GR2E rice was found to have a 23 bp deletion at the right border end and an 11 bp deletion at the left border end, which is common for *Agrobacterium*-mediated transformation events^20^. All remaining sequence was intact and identical to that of the T-DNA region of plasmid pSYN12424.

**Figure 2:**
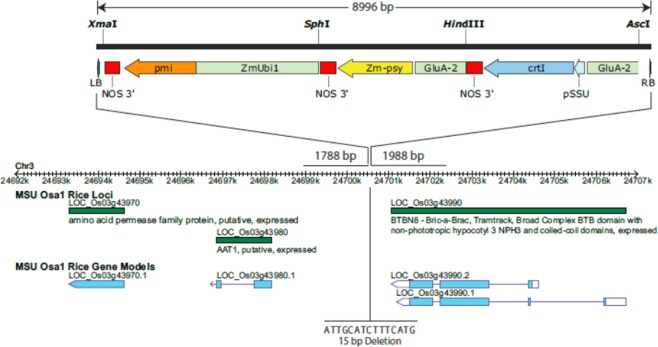
Map position is indicated according to the MSU Rice Genome Annotation Project Release 7 (Nipponbare). The locations of the LB and RB flanking sequences correspond to positions 24,698,762–24,700,549 and 24,700,565–24,702,552, respectively. The insertion of the pSYN12424 T-DNA was within an intergenic region between loci LOC_Os03g43980 and LOC_Os03g43990, and resulted in the deletion of 15 bp of host genomic DNA in addition to truncations of the LB and RB regions of 11 bp and 23 bp, respectively (adapted from GR2E-FFP submitted study reports).

Basic local alignment search tool searches using the 5′ and 3′ flanking region sequences as queries against the *O. sativa* (japonica cultivar-group, Nipponbare) genome (MSU Rice Genome Annotation Project Release 7) localized the T-DNA on chromosome 3 within the intergenic region between LOC_Os03g43980 (3′ proximal) and LOC_Os03g43990 (5′ proximal; Fig. 2, adapted from GR2E-FFP submitted study reports).

To investigate the possibility of creating new ORFs as a consequence of the T-DNA insertion in GR2E, an open reading frame analysis was conducted to look for potential start-to-stop ORFs that spanned either the 5′ or 3′ junctional regions. This analysis examined each of three possible reading frames in both orientations (i.e., six possible reading frames in total) for potential ORFs capable of encoding sequences of 30 or more amino acids. An allergen usually contains at least two epitopes, each of which will be a minimum of approximately 15 amino acid residues long, in order that antibody binding could occur. This implies a lower size limit for protein allergens of approximately 30 amino acid residues^21^, although currently there is no consensus among scientist on such size limit. Two ORFs were identified, one in the reverse orientation that spanned the 5′ T-DNA insert–genomic DNA border (Supplementary Fig. 4, adapted from GR2E-FFP submitted study reports; ORF-1, 207 bp, 68 amino acids), and one in the forward orientation that spanned the 3′ T-DNA insert–genomic DNA border (Supplementary Fig. 4, adapted from GR2E-FFP submitted study reports; ORF-2, 240 bp, 79 amino acids).

To search for potential similarity to known toxins, the amino acid sequence of each ORF was queried against a toxin database using the FAST All sequence alignment tool 36 (FASTA36) to identify possible significant sequence similarity with known or potential toxins. An *E*-score criterion of 1 × 10^−5^ was used to identify sequences from the toxin database with potential for significant sequence similarity to the query sequences of each ORF. Typically, alignments between two sequences require an E-score of 1 × 10^−5^ or less to be considered to have sufficient sequence similarity to infer homology. The FASTA36 search resulted in no significant hits returned (Supplementary Table 6).

To assess the potential for allergenicity, the amino acid sequence of each ORF was compared to a peer-reviewed database of 2129 known and putative allergens and celiac protein sequences residing in the Food Allergy Research and Resource Program (FARRP) dataset version 19 at the University of Nebraska. A criteria of > 35% identity over any segment of 80 or more amino acids as an indication of possible cross-reactivity for allergens was adopted by the Codex^12^ as the primary sequence search criteria for use in flagging proteins that might be of some concern of cross-reactivity for genetically modified plants. No identity matches of greater than 35 percent over 80 residues were observed for either ORF-1 or ORF-2. Each query sequence was also evaluated for any eight contiguous identical amino acid matches to the allergens contained in the FARRP database. There were no eight contiguous identical amino acid matches observed for either ORF-1 or ORF-2 (Supplementary Table 6).

### Novel protein expression

The tissue specificity of *Zm*PSY1, CRTI, and PMI expression was confirmed by immunoblot analysis of various tissues sampled from GR2E rice. The patterns of expression of proteins in GR2E tissues were consistent with the activity of the endosperm-specific rice *GluA-2* promoter of the *Zmpsy1* and *crtI* genes, and use of the constitutive maize polyubiquitin promoter for the *pmi* gene. Expression of *Zm*PSY1 and CRTI was detected only in milk, dough, and mature stage grain (Supplementary Fig. 5, lanes 3–5, panels B and D, adapted from GR2E-FFP submitted study reports) and not in samples of bran, hulls, leaf, stem, or root tissue. In comparison, PMI expression was detected in all rice tissues tested (Supplementary Fig. 5, lanes 3–10, panel F, adapted from GR2E-FFP submitted study reports).

In order to estimate potential human and animal dietary exposure to the *Zm*PSY1, CRTI, and PMI enzymes expressed in GR2E, the protein concentrations in plant tissues were determined by quantitative enzyme-linked immunosorbent assay (ELISA). Three replicated samples of grains (milky, dough, mature) and straw were collected from GR2E rice grown at four locations in the Philippines during two growing seasons in 2015–16. Expression of the *Zm*PSY1 and CRTI proteins in GR2E is driven by the endosperm-specific rice *GluA-2* promoter and measurable concentrations of both these proteins were found in all grain developmental stages but not in stem tissue (straw; Supplementary Table 7). For each protein, the highest concentrations were measured in samples of dough-stage grain, ranging between *ca*. 308–359 ng/g fresh weight tissue (FWT) and between ca. 54–68 ng/g FWT for *Zm*PSY1 and CRTI, respectively, across both growing seasons. Across the four locations and two growing seasons, the highest concentrations of *Zm*PSY1 and CRTI measured in samples of mature grain were *ca*. 245 ng/g FWT and 30 ng/g FWT, respectively.

**Figure 3:**
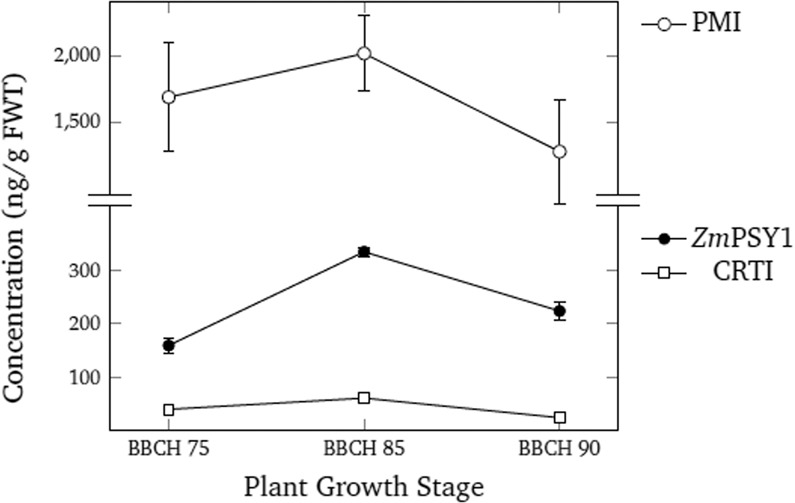
Samples of GR2E grain were collected at different developmental stages [BBCH 75 (milk stage), BBCH 85 (dough stage), and BBCH 90 (mature stage)] from four locations over two growing seasons in 2015–16 and the concentrations of ZmPSY1, CRTI, and PMI were determined by quantitative ELISA and are given in ng g/fresh weight tissue (FWT). Values represent the mean concentration across locations and years for each protein, and the error bars represent the range of concentrations measured across locations over both growing seasons. In some cases, the size of the error bars was less than the symbol size used for plotting (adapted from GR2E-FFP submitted study reports).

Concentrations of PMI protein were significantly higher than either *Zm*PSY1 or CRTI in samples from all grain growth stages (Fig. 3, adapted from GR2E-FFP submitted study reports), and were highest in dough-stage grain, averaging *ca*. 2015 ng/g FWT across the four locations over both growing seasons. The mean PMI concentration in mature GR2E rice grain samples was *ca*. 1282 ng/g FWT across both growing seasons (Supplementary Table 7). Since expression of the PMI protein was under control of the constitutive maize polyubiquitin promoter, it was also present in straw samples at concentrations ranging between 320–796 ng/g FWT depending on location and growing season. The average concentration of PMI protein in GR2E straw across both growing seasons was *ca*. 482 ng/g FWT.

### Estimated human daily dietary exposure to ZmPSY1, CRTI, and PMI proteins

Two approaches were followed to obtain estimates of daily rice consumption. First, historic rice utilization data for the highest rice-consuming countries in Asia, in comparison with the United States, were obtained from the USDA Production Supply and Distribution database and converted to per capita utilization estimates using the FAOSTAT population database. These values for 2011–2015 are presented in Supplementary Table 8. Projected utilization values for the same countries were obtained from the International Rice Outlook: International Rice Base Projections^22^, also presented in Supplementary Table 8 and Supplementary Fig. 6 (adapted from GR2E-FFP submitted study reports).

Using the highest projected per capita rice utilization in Cambodia of 253 kg/yr and an estimated average adult body weight of 57.7 kg in Asia^23^, the maximum daily rice intake was calculated as shown in this equation:

$${\rm{Daily}}\,{\rm{Rice}}\,{\rm{Intake}}=\frac{253\,({\rm{kg}}/{\rm{yr}})}{365\times 57.7\,({\rm{kg}}\,{\rm{BW}})}{\rm{X}}\,1000\,({\rm{g}}/{\rm{kg}})=12.0\,({\rm{g}}/{\rm{kg}}\,{\rm{body}}\,{\rm{weight}})$$

The second approach utilized data from the Food and Agriculture Organization of the United Nations (FAO)/World Health Organization (WHO) Chronic Individual Food Consumption Database summary statistics (CIFOCOss) currently containing summary statistics of 37 surveys from 26 countries. The CIFOCOss was initially developed to be used by FAO/WHO scientific committees for dietary exposure assessment. Available data for Asian countries are shown in Supplementary Table 9. A further comparison of consumption data between Asian countries and selected European, African, and South American countries is shown in Supplementary Fig. 7 (adapted from GR2E-FFP submitted study reports).

Based upon consideration of both approaches, a value of 12.5 g/kg body weight was chosen as the upper limit of mean daily dietary intake of rice. This value was judged as sufficient to account for consumption by all population subgroups, including children. In deriving estimates of maximum potential daily dietary exposure to the *Zm*PSY1, CRTI, and PMI proteins expressed in GR2E rice, the following assumptions were used: (i) The mean daily dietary rice consumption is 12.5 g/kg body weight, (ii) 100% percent of the dietary rice intake is from GR2E rice and (iii) the grain concentrations of *Zm*PSY1, CRTI, and PMI used for estimation are the highest values measured. This is the case in samples of dough-stage grain collected from any individual trial site location in either 2015 or 2016. These concentrations were significantly higher than those measured in mature grain at harvest. Using these assumptions, the estimated maximum potential daily dietary exposure from GR2E rice to each novel protein is shown in Table 2. They are estimated to be *ca*. 4.5, 0.85, and 30 μg/kg body weight to *Zm*PSY1, CRTI, and PMI proteins, respectively.

**Table 2:** Estimated maximum potential daily dietary exposure to *Zm*PSY1, CRTI, and PMI.

Protein	Concentration (ng/g FWT)	Daily Dietary Exposure (µg/kg body weight)^†^
*Zm*PSY1	359	4.49
CRTI	68	0.85
PMI	2397	29.96

^†^Daily dietary exposures (µg/kg body weight) to *Zm*PSY1, CRTI, and PMI were calculated based on daily *per capita* rice consumption of 12.5 g/kg body weight.

### Bioinformatic analysis of the ZmPSY1 and CRTI protein amino acid sequences

PSY plays a pivotal role in the carotenoid biosynthesis pathway as it catalyzes the first committed step and controls flux through the pathway^24,25^. Phytoene undergoes consecutive modifications such as desaturation reactions by carotene desaturases and cis-trans isomerization reactions to form all-trans-lycopene, which is cyclized to α- and β-carotene.

Potential identities between the *Zm*PSY1 query sequence and proteins in the allergen database were evaluated with the FASTA35 sequence alignment tool using the default parameters. A criteria of > 35% identity over any segment of 80 or more amino acids as an indication of possible cross-reactivity to allergens was adopted by the Codex^12^ as the primary sequence search criteria for use in flagging proteins that might be of some concern of cross-reactivity for genetically modified plants. No identity matches of > 35 percent over 80 residues were observed. Also, there were no instances of eight contiguous identical amino acid matches observed between the amino acid sequence of *Zm*PSY1 when compared with the sequences of known allergenic proteins. To search for similarity to known or potential toxins, the amino acid sequence of the *Zm*PSY1 was queried against a toxin database using the FASTA36 algorithm. The *Zm*PSY1 query sequence did not return any entries with *E*-*score* less than 1 × 10^−5^. Therefore, there are no sequence homology alerts for potential toxicity of the *Zm*PSY1 protein.

Potential identities between the CRTI query sequence and proteins in the allergen database were evaluated and no identity matches of > 35 percent over 80 residues were observed, nor were there any instances of eight contiguous identical amino acid matches observed between the CRTI amino acid sequence and sequences of known allergenic proteins. However, a search using the CRTI query sequence returned two protein accessions from the toxin database with an *E-score* less than 1 × 10^−5^. The two sequence alignments (Supplementary Fig. 8, adapted from GR2E-FFP submitted study reports) were to the conserved N-terminal FAD (flavin adenine dinucleotide) -binding regions of L-amino acid oxidase (LAAO) enzymes from two species of venomous snakes: *Bungarus multicinctus* (many-banded krait, also known as the Taiwanese krait or the Chinese krait) and *B. fasciatus* (banded krait). Homology of these proteins will be discussed later.

### Rapid digestion of ZmPSY1 and CRTI in simulated gastric fluid (SGF)

Rapid gastric and intestinal digestion is known to be correlated to the allergenic potential of proteins^26^. The *in vitro* pepsin resistance of native i.e. enzymatically active *Zm*PSY1 protein was investigated. Samples were removed at the given stated time points and subjected to SDS-PAGE analysis. Following exposure to SGF-containing pepsin for 30 seconds, the earliest time point sampled during the digestion, no intact *Zm*PSY1 protein (*ca*. 42 kDa) was evident as assessed by either SDS-PAGE or western immunoblot analysis (Supplementary Fig. 9, lane 4 in panels A and B, respectively, adapted from GR2E-FFP submitted study reports). Faint, low molecular mass degradation products were visible by Coomassie staining in samples removed up to two minutes of digestion (Supplementary Fig. 9, lanes 4–6, panel A, adapted from GR2E-FFP submitted study reports), but not at later time points, and these were not detected in the western blot.

**Figure 4:**
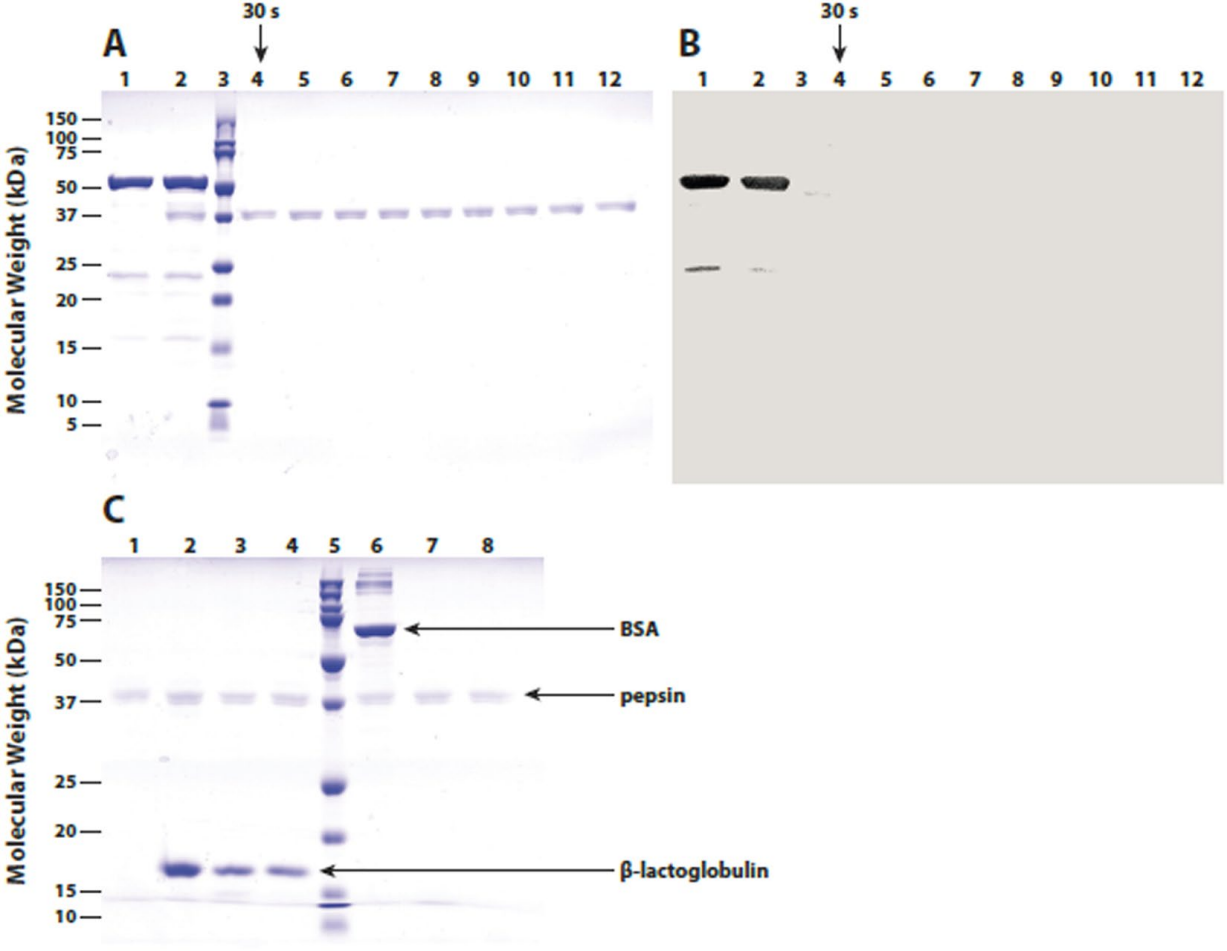
Panels A and B: Samples of CRTI protein purified from recombinant E. coli (Lot No. M20454-02) were incubated in the presence of SGF pH 1.2 containing pepsin for 0 min (lane 2) and 0.5, 1, 2, 5, 10, 20, 30 or 60 min at 37 °C (lanes 4–11) and then analyzed by SDS-PAGE. Gels were either stained for protein with colloidal blue G250 (panel A) or subjected to western immunoblot analysis (panel B) using rabbit anti-CRTI immunoglobulin (1:1000) and horseradish peroxidase-conjugated goat anti-rabbit IgG followed by precipitating substrate development. Control samples included CRTI protein diluted in gastric control fluid without pepsin (lane 1) and SGF solution containing pepsin (lane 12). Molecular weight standards are shown in lane 3 (adapted from GR2E-FFP submitted study reports).

Similar results were obtained with CRTI. Following exposure to SGF containing pepsin for 30 seconds, the earliest time point sampled during the digestion, no intact CRTI protein was evident as assessed by either SDS-PAGE or western immunoblot analysis (Fig. 4, lane 4 in panels A and B, respectively, adapted from GR2E-FFP submitted study reports), and there was no evidence of stable lower molecular mass proteolytic fragments derived from CRTI.

### Heat stability of ZmPSY1 and CRTI protein

**Figure 5:**
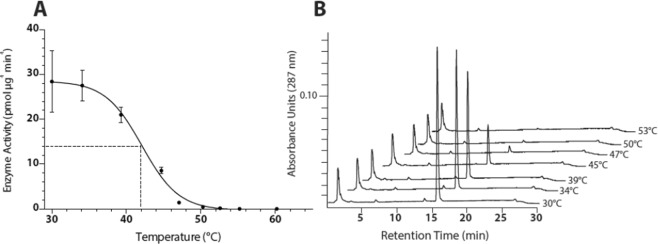
Individual samples of ZmPSY1 protein purified from recombinant E. coli (Lot No. M20452-05) were heated for 15 minutes at a designated temperature ranging from 30–65 °C. Following this treatment, enzymatic production of 15-cis-phytoene was measured by HPLC. Panel A shows enzymatic activity (pmol μg-1 min-1) versus pre-incubation temperature, where the values are means + /− standard deviation of two technical replicates. Panel B shows HPLC chromatograms (287 nm) of chloroform:methanol extracts of selected ZmPSY1 activity assays. The area under the phytoene peak (retention time = 15.7 min) was used to calculate phytoene concentration. Pre-incubation temperatures are indicated to the right of each chromatogram trace (adapted from GR2E-FFP submitted study reports).

The thermal stability of the *Zm*PSY1 protein was evaluated by measuring enzymatic activity, i.e. the conversion geranylgeranyl diphosphate (GGPP) into 15-*cis*-phytoene, as monitored by HPLC analysis. The GGPP substrate was produced with GGPP-synthase from its precursor molecules dimethylallyl diphosphate (DMAPP) and isopentenyl diphosphate (IPP)^24,27^. Thermal instability, i.e. rapid denaturation greatly increases the chance for proteolytic cleavage, adding to the safety of the expressed proteins. Proteins that are labile to temperatures used in cooking and processing are likely to have negligible dietary exposure. The expressed purified *Zm*PSY1 catalyzed the production of 15-*cis*-phytoene from DMAPP and IPP, in the presence of active *A. thaliana* GGPP synthase, at the rate of ca. 28.4 pmol *μg*^−1^ min^−1^ under the assay conditions used (Fig. 5, adapted from GR2E-FFP submitted study reports). Enzyme activity was irreversibly destroyed upon heat treatment, with 50 percent loss of activity following pre-incubation at *ca*. 42 °C for 15 minutes and complete loss of activity at 50 °C for 15 minutes.

The thermal stability of the CRTI protein was evaluated by measuring enzymatic activity using a spectrophotometric assay to monitor the conversion of liposome-incorporated 15-*cis*-phytoene to all-*trans*-lycopene according to assay conditions^28^. The purified CRTI was enzymatically active, catalyzing the conversion of liposome-incorporated phytoene to all-*trans*-lycopene at the rate of *ca*. 5.4 pmol all-*trans*-lycopene *μg*^−1^ min^−1^ under the assay conditions used (Supplementary Fig. 10, adapted from GR2E-FFP submitted study reports). Enzyme activity was irreversibly destroyed upon heat treatment, with 50 percent loss at *ca*. 51 °C for 15 minutes and complete loss of activity following pre-incubation at 55 °C for 15 minutes.

### Lack of acute toxicity of CRTI protein

The potential for acute toxicity resulting from a single oral exposure to CRTI was investigated in mice. Groups of five male and five female mice were dosed orally by gavage with: formulation buffer; bovine serum albumin or purified microbial-expressed CRTI (100 mg/kg body weight actual dose), at a volume of 13.45 ml/kg body weight, administered in two separate doses approximately 4 hours apart on test day 1. All animals survived until the scheduled end of the study period on day 15 and there were no clinical signs (abnormal behavior, general appearance and mortality/moribundity) of toxicity observed during the test period, nor were any gross lesions found in the mice at necropsy. There were no treatment-related effects on body weights for male or female mice over the study duration and all mice experienced net weight gain by test day 15 compared with test day 1 (pre-fast).

## Discussion

The purpose of this evaluation of GR2E rice was to determine whether the use of GR2E rice could raise any safety concerns relative to conventional rice. This assessment was not intended to address questions related to the efficacy of GR2E rice in helping combat VAD in affected population sub-groups.

The characterization of the inserted T-DNA by Southern blot analyses and nucleotide sequencing demonstrated that the inserted expression cassettes for the *Zmpsy1*, *crtI*, and *pmi* genes were intact and without sequence changes. They were introduced at a location in the genome that is unlikely to affect the expression of any endogenous genes. Moreover, the two novel ORFs that were created were unlikely to have the potential to encode toxins or allergens. The inserted genes and production of carotenoids were stably inherited across multiple generations according to Mendelian patterns of segregation, consistent with a single locus.

In the pepsin digestion, heat stability assay and ELISA, both *Zm*PSY1 and CRTI used were microbial-expressed proteins. Based on the combination of physiochemical (N-terminal sequence and mass spectral analyses) and enzyme activity analyses, both proteins were functionally equivalent to the *in planta* expressed counterpart, and were suitable surrogate proteins for conducting relevant safety studies.

*Zea mays* (maize) was the source of the *psy1* gene^29^ Maize, a food crop with a long history of safe use, is cultivated worldwide as the third most planted crop after wheat and rice. No significant endogenous toxins are reported to be associated with the genus *Zea*^30^. Food allergy to maize is relatively rare, and the only significant reported food allergen is a nonspecific lipid transfer protein^31^. The *Zmpsy1* gene does not have amino acid sequence similarity to known allergenic and toxic proteins, is not resistant to *in vitro* gastric digestion, and is heat labile. This resulted in the conclusion that further hazard characterization by animal toxicity testing was unnecessary.

*Pantoea ananatis* was the source of the *crtI* gene^32^ and is found in a wide range of natural environments, including water, soil, as part of the epi- and endo-phytic flora of various plant hosts^33^. *Pantoea ananati*s is a ubiquitous bacterium which is found on fresh fruit and vegetables^34^. Many members of the *Enterobacteriaceae*, belonging to the genera *Serratia, Enterobacter, Pantoea, Proteus*, and *Hafnia*, often contribute to meat spoilage^35^. The ubiquity of *P. ananatis* suggests that it has adapted to proliferate in a wide range of environments, and its isolation from both plant and animal hosts indicate that it has adapted for cross-kingdom colonization and pathogenesis^36^. Although strains of *P. ananatis* have been found to be pathogenic on a broad range of plant hosts as well as humans, *crtI* is not part of the genomic material that is associated with the organism’s pathogenicity and virulence. There is a possibility of some dietary exposure to CRTI protein at low levels as a consequence of adventitious presence of *P. ananatis* on foods. However, a history of dietary exposure is difficult to establish.

Due to the non-food source of the *crtI* gene, acute oral toxicity testing of CRTI protein in mice was conducted as an additional assurance of safety. Oral administration of CRTI test substance at a concentration representing at least an 115,000-fold margin of exposure relative to any realistically conceivable human dietary intake produced no test substance-related clinical signs of toxicity, body weight losses, gross lesions, or mortality, further substantiating the predicted lack of acute oral toxicity of CRTI. Additionally, CRTI does not have amino acid sequence similarity to proteins known to be allergens or toxic via the oral route of exposure and was readily degraded in the presence of pepsin and inactivated when exposed to heat. A 90 day feeding trial to rodents was not mandatory for approval in the target countries and in some regulatory agencies.

The limited sequence similarity between the *P. ananatis* CRTI protein and the LAAO accessions retrieved from the toxin database was due to homology between N-terminal motifs involved in FAD (flavin adenine dinucleotide) binding, and was not considered to be a structural alert for potential toxicity. LAAOs are flavoenzymes belonging to the class of oxidoreductases that catalyze the stereospecific oxidative deamination of L-amino acids, and snake venom LAAOs are usually homodimeric with cofactors FAD or FMN (flavin mononucleotide) covalently linked to their chemical structure. CRTI also belongs to the flavoprotein superfamily comprising, for instance, protoporphyrinogen IX oxidoreductase and monoamine oxidase and has an absolute requirement for FAD as the sole cofactor in CRTI-mediated phytoene desaturation. Thus, the limited sequence similarity between the CRTI protein and the two L-amino acid oxidase accessions is not surprising and is due to homology between N-terminal motifs involved in FAD binding. Indeed, similar homologies exist between the native rice phytoene desaturase (*Os*PDS) and LAAOs. The two desaturases, CRTI and *Os*PDS, have likely evolved resulting in two different approaches to achieving similar catalytic goals^28^.

In summary, the molecular-genetic characterization of GR2E rice, including the assessment of potential toxic or allergic reaction to the newly expressed *Zm*PSY1 and CRTI proteins, when considered together with the comparative compositional assessment^10^, support the conclusion that food derived from rice varieties containing event GR2E is as safe as food derived from conventional rice varieties. All the data presented in the regulatory dossier is mainly intended to meet the regulatory requirements in Philippines, Bangladesh, and Indonesia. Other countries may have other requirements.

## Methods

### Molecular genetic characterization of GR2E rice

Genomic DNA extraction was performed as described by Murray and Thompson^37^. For Southern analyses, genomic DNA samples extracted from selected homozygous GR2E and control rice plants were digested with *Asc*I + *Xma*I to investigate the integrity of the inserted T-DNA, and with either *Hin*dIII or *Sph*I to determine the number of copies of the inserted DNA. Samples were prepared from GR2E in four different germplasm backgrounds: Kaybonnet, BRRI *dhan* 29, IR64, and PSB Rc82. The stability of the inserted DNA across multiple generations of GR2E rice was assessed by DNA blot analyses of genomic DNA samples prepared from the Tn generation and the BC_3_F_5_, BC_4_F_3_, and BC_5_F_3_ generations of GR2E in BRRI *dhan* 29, IR64, and PSB Rc82 germplasm. Probe DNA was synthesized by PCR according to the procedures supplied in the PCR DIG Probe Synthesis Kit (Roche). Probes for the *Zmpsy1*, *pSSU-crtI*, and *pmi* genes were used to detect genetic elements within the insertion. Probes covering the backbone region of plasmid were used to verify absence of plasmid backbone DNA in GR2E rice (Supplementary Fig. 11, adapted from GR2E-FFP submitted study reports). DNA fragments of the probe elements were generated by PCR from plasmid using specific primers (Supplementary Table 1).

### Segregation analysis

Analyses were performed on individual plants from three segregating generations representing the BC_4_F_2_, BC_5_F_1_, and BC_5_F_2_ generations of GR2E rice in PSBRc82, BRRI dhan 29, or IR64 genetic background. DNA samples were analyzed by multiplex PCR to determine zygosity of the T-DNA insert as illustrated in Supplementary Fig. 12 (adapted from GR2E-FFP submitted study reports). Individual PCR reaction mixtures contained 300 ng genomic DNA, 0.5 µΜ of each primer (ZD-E1-P1, ZD-E1-P2, and Zd-E1-P3), 0.2 mM deoxynucleotide triphosphates, 1.5 mM MgCl_2_, and 0.5 units KAPA Taq DNA polymerase (KAPA Biosystems) in 1x KAPA Taq buffer. After an initial 5 min denaturation at 95 °C, PCR amplifications were performed for 35 cycles consisting of denaturation (95 °C, 45 sec), annealing (55 °C, 45 sec), and extension (72 °C, 45 sec). A final extension step (72 °C, 8 min) was included following the last PCR cycle, after which samples were held at 25 °C. Following PCR, reaction mixtures were analyzed by agarose gel electrophoresis in 1.5 percent agarose and amplified fragments were visualized with SYBR® Safe DNA Gel Stain (Thermo Fisher Scientific). A Chi-square analysis was performed on the segregation results of each GR2E rice generation to compare the observed segregation ratio to the expected Mendelian segregation ratio. The critical value to reject the hypothesis at the 5 percent level is 3.84.

### Nucleotide sequence analysis of GR2E rice

The procedures for cloning the DNA fragments from GR2E and its sequencing are described in Supplementary Method 1.

### Enzyme-linked immunosorbent assay

Samples of rice grain were collected at the milk, dough, and mature stages of development^38^. Each individual sample was a composite of material obtained from at least five representative plants. Approximately 3–4 panicles were collected from each representative plant and placed in a pre-labelled net bag, one per block. Following collection of all plants, grains for each sample and growth stage was removed from the panicles, mixed, and ca. 100 g placed in 50 ml screw-top Falcon tubes. Samples of rice straw were collected at harvest. Each individual sample was a composite of material obtained from at least five representative plants. Following collection, straw was chopped into small pieces ca. 12 cm in length, mixed, and ca. 100 g placed in a pre-labelled sample bag. Concentrations of the ZmPSY1, CRTI, and PMI proteins were determined using specific quantitative ELISA methods (GR2E-FFP submitted study reports).

### Immunoblot analysis of ZmPSY1, CRTI, and PMI expression in different plant tissues

Frozen tissue samples were ground to a powder in liquid nitrogen using a mortar and pestle. Weighed amounts (200 mg) were re-suspended in either 950 μl (grain, bran, and hulls) or 425 μl (stem, leaf, and root tissue) of 1 × Laemmli sample buffer containing 350 mM dithiothreitol (DTT), vortexed (1 min), and placed on ice for 30 minutes. Tissue extracts were centrifuged (12000 g, 10 min at 4 °C) and supernatant fractions were transferred to new 1.5-ml tubes. The total protein concentration of each sample was determined using the bicinchoninic acid (BCA) protein assay kit (Pierce, Thermo Scientific). Sample extracts containing either 40 μg (ZmPSY1 and CRTI blots) or 7 μg (PMI blots) total protein were subjected to sodium dodecylsulfate polyacrylamide gel electrophoresis (SDS-PAGE) on 10 percent Tris-glycine polyacrylamide gels at 40 V for 30 min followed by 50 V for three hours. For each sample set, one gel was stained with colloidal blue G250 and another was electroblotted onto polyvinylidene fluoride (PVDF) membrane for immuno-labelling. Each sample set contained a positive control sample consisting of non-transgenic Kaybonnet dough-stage grain extract spiked with either purified ZmPSY1 (M20452-05; 2.5 ng), CRTI (M20454-02; 25 ng), or PMI (21038 G; 6.25 ng), and a negative control sample of non-transgenic Kaybonnet dough-stage grain extract.

Membrane blots were incubated for 30 min at room temperature in Tris-buffered saline pH 7.4, 0.05% Tween-20 (TBST) containing 5 percent non-fat dry milk (NFDM) to block non-specific protein binding sites. Membranes were then incubated with either mouse monoclonal antibody specific for ZmPSY1 (2 μg/ml; ELX1048, Envirologix) or CRTI (2 μg/ml; ELX1043, Envirologix) overnight at 4 °C with shaking followed by rinsing with five changes of TBST (5 min each). Rinsed blots were incubated with alkaline phosphatase (AP)-conjugated goat anti-mouse IgG (1:10,000 in TBST + 5% NFDM, 120 min) followed by rinsing as before with TBST. Bound antibody-conjugate was detected using CDP-Star chemiluminescent substrate (Roche) and exposure on Kodak Biomax MS film followed by electronic image capture using a white light transilluminator (Vilber Lourmat, France). For detection of PMI protein, blocked membranes were incubated with horseradish peroxidase (HRP)-conjugated rabbit anti-PMI antiserum (1:100; Romer Labs) followed by chemiluminescent substrate development using luminol substrate (GE Healthcare, USA).

### Amino acid sequence similarity search between proteins and known and putative protein toxins and allergens

A FASTA36 bioinformatic search using the ZmPSY1 and CRTI amino acid sequences as the query sequence was performed against a toxin database to identify possible significant sequence similarity with known or potential toxins. The toxin database was created from a subset of sequences derived from the UniProt Knowledgebase, comprised of 550,116 manually annotated and reviewed sequences from Swiss-Prot and 55,270,679 automatically annotated, un-reviewed sequences from TrEMBL^39^, that were selected using a keyword search on toxins (KW800). The collection contained a total of 24,098 sequences as of 21 January 2016, comprising 6,588 reviewed sequences from Swiss-Prot and 17,510 un-reviewed sequences from TrEMBL. The BLOSUM50 similarity scoring matrix was used for FASTA36 alignments^40^. An E-score acceptance criterion of 1 × 10^−5^ was used to identify sequences with potential for significant sequence similarity to the proteins.

To assess the potential for allergenic cross-reactivity, the amino acid sequence encoded by the *Zmpsy1* and *crtI* genes was compared to a database of 2129 known and putative allergen and celiac protein sequences residing in the FARRP19 dataset at the University of Nebraska. Potential identities between the query sequence and proteins in the allergen database were evaluated with the FASTA35 sequence alignment tool using the default parameters. The recommended 35 percent or greater identity threshold over any 80-amino acid length sequence alignment between the query sequence and an allergen was used to indicate the potential for cross-reactivity.

Sequence similarity searches to known Allergens using ORF-1 and ORF-2 sequences as queries are described in Supplementary Method 2.

### *In vitro* pepsin digestions

The *in vitro* pepsin resistance of ZmPSY1 and CRTI protein were investigated by incubating purified ZmPSY1 and CRTI protein for 0, 0.5, 1, 2, 5, 10, 20, 30, and 60 minutes at 37 °C in SGF pH 1.2 containing pepsin. Control digestions with bovine serum albumin (BSA) and beta-lactoglobulin were performed for 0, 1, and 60 minutes under the same conditions. Samples were removed at stated time points and subjected to SDS-PAGE and immunoblot analyses.

### Heat stability of ZmPSY1 and CRTI protein

The thermal stability of the ZmPSY1 protein was evaluated by measuring enzymatic activity using a HPLC method to monitor the production of 15-*cis*-phytoene from *in situ* produced geranylgeranyl diphosphate (adapted from GR2E-FFP submitted study reports). Samples of microbial-expressed, purified ZmPSY1 protein were subjected to heat treatment over a temperature incubation range of ca. 30–65 °C for 15 minutes and then used in activity assays. Chloroform extracts of individual reaction mixtures were separated by reverse-phase HPLC and ZmPSY1 enzyme activity was calculated based on phytoene peak area measurements.

The thermal stability of CRTI protein was evaluated by measuring enzymatic activity using a spectrophotometric assay to monitor the conversion of liposome-incorporated 15-*cis*-phytoene to all-*trans*-lycopene. Samples of microbial-expressed, purified CRTI protein (6 μg) were subjected to heat treatment over a temperature incubation range of ca. 30–60 °C for 15 minutes, following measurement of enzyme activity at 37 °C in the presence of 7 μM phytoene, 150 μM flavin adenine dinucleotide, 50 mM Tris-HCl pH 8.0, and 200 mM NaCl.

### Acute toxicity of CRTI protein

Groups of five male and five female Crl:CD1(ICR) mice were dosed orally by gavage with: formulation buffer (vehicle control; 50 mM Tris-HCl pH 8.0, 200 mM NaCl, 1 mM tris(2-carboxyethyl)phosphine (TCEP)); bovine serum albumin (BSA; negative control; target dose 100 mg/kg body weight dissolved in vehicle control solution); or purified microbial-expressed CRTI (100 mg/kg body weight actual dose purified protein dissolved in vehicle control solution), at a volume of 13.45 ml/kg body weight, administered in two divided doses ca. 4 hours apart on test day 1. Body weights were evaluated on test days 1 (pre-fast and shortly prior to administration of the first dose), 2, 3, 5, 8, and 15. Clinical signs (abnormal behavior, general appearance and mortality/moribundity) were evaluated seven times on test day 1 (distributed before and after each dose) and daily thereafter. On test day 15, all mice were euthanized and subject to a gross pathological examination.

### GR2E provitamin A biofortified rice regulatory dossier

The complete package of GR2E provitamin A biofortified rice regulatory dossier submissions can be found at https://www.dropbox.com/sh/qpiz0cftefcaceq/AAByIpj_HED3zgqH7ufW7A-ta?dl=0^41^; https://www.foodstandards.gov.au/code/applications/Documents/A1138%20Application_Redacted.pdf^42^

## Supplementary information

Supplementary information

## Data Availability

All the data has been provided in the manuscript.
