# Supplementary material for: Molecular characterization and safety assessment of biofortified provitamin A rice
Source: Sci Rep. 2020 Jan 28;10:1376. doi: 10.1038/s41598-020-57669-5 (PMC6987151; doi:10.1038/s41598-020-57669-5)
Supplement: Supplementary file 1 — Supplementary information [file 41598_2020_57669_MOESM1_ESM.pdf]

Molecular characterization and safety assessment of biofortified provitamin A rice

Norman Oliva, Maria Florida Cueto-Reaño, Kurniawan Trijatmiko, Mercy Samia, Ralf Welsch, Patrick Schaub, Peter Beyer, Donald Mackenzie, Raul Boncodin, Russell Reinke, Inez Slamet-Loedin, B.P. Mallikarjuna Swamy

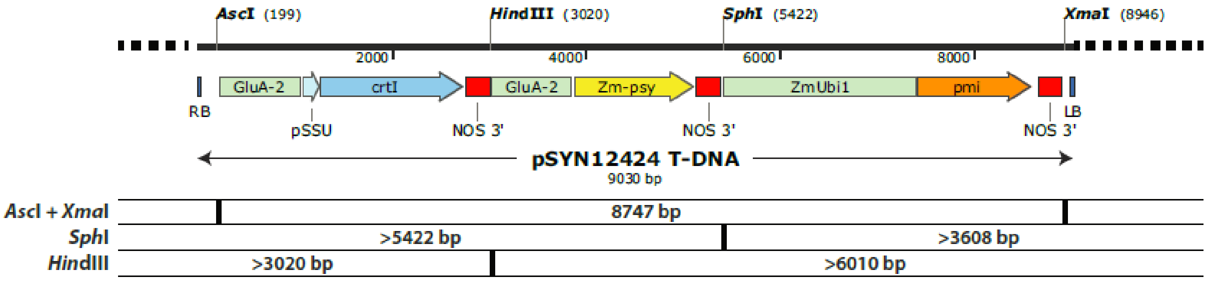

Supplementary Fig. 1 Schematic map of the pSYN12424 T-DNA showing the *Asc*I, *Hin*dIII, *Sph*I, and *Xma*I restriction endonuclease sites used for Southern Hybridization characterization of GR2E rice. The flanking rice genome is represented by the horizontal dotted line. Based on the insertion of a single copy of the T-DNA at a single site, the predicted sizes of restriction fragments following digestion with *Asc*I plus *Xma*I (8747 bp), *Sph*I (>5422 bp and >3608 bp), or *Hin*dIII (>3020 bp and >6010 bp) are shown. Numbering shown on the map is relative to the pSYN12424 T-DNA (adapted from GR2E-FFP submitted study reports)

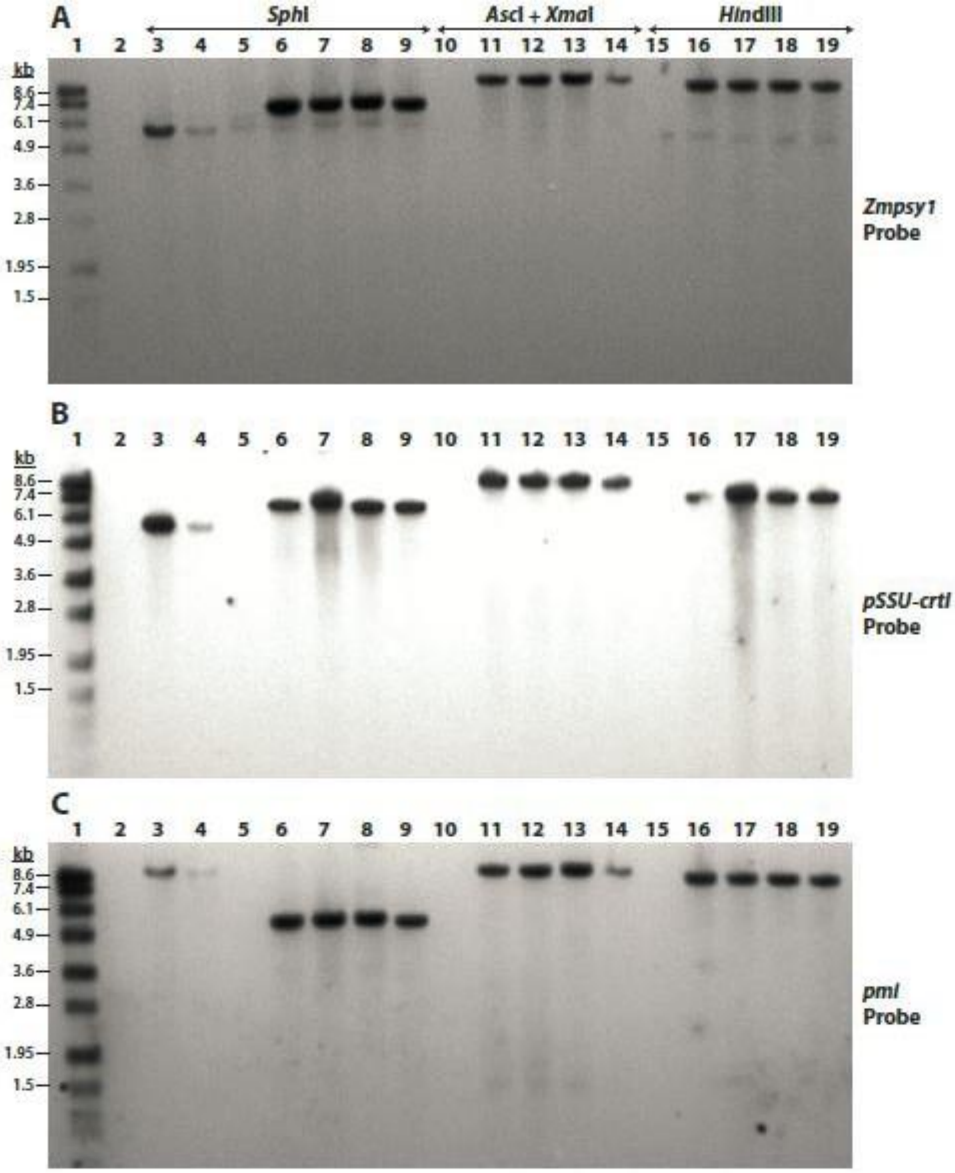

Supplementary Fig. 2 Samples of genomic DNA (ca. 7 µg) from individual plants of event GR2E in Kaybonnet (Tn; lanes 6, 11, and 16), BR29 (BC_5F_3; lanes 7, 12, and 17), IR64 (BC_5F_3; lanes 8, 13, and 18), and PSB Rc82 (BC_5F_3; lanes 9, 14, and 19) germplasm backgrounds; negative control DNA from Kaybonnet rice (lanes 5, 10, and 15); and negative control Kaybonnet rice containing either ca. one (lane 3) or 0.2 (lane 4) copy equivalents of pSYN12424 plasmid DNA, were subjected to restriction endonuclease digestion with *Sph*I (lanes 3–9), *Asc*I plus *Xma*I (lanes 10–14), or *Hin*dIII (lanes 15–19) followed by agarose gel electrophoresis and transfer onto nylon membrane. Blots were hybridized with DIG-labelled probes specific for *Zmpsy1* (panel A), *SSU-crtI* (panel B), or *pmi* (panel C). Following washing, hybridized probes and DIG-labelled molecular weight markers VII (lane 1) were visualized using a chemiluminescent detection system followed by electronic image capture. Lane 2 was blank on all gels (adapted from GR2E-FFP submitted study reports)

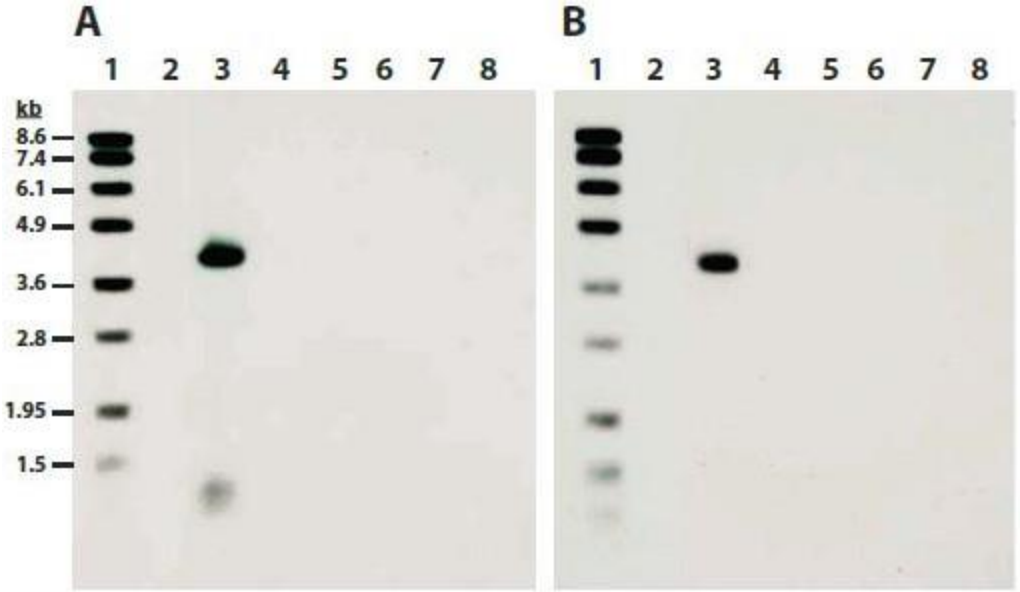

Supplementary Fig. 3 Samples of genomic DNA (ca. 7 µg) from control Kaybonnet rice (lanes 4 and 5), event GR2E in Kaybonnet (Tn; lanes 6–8), and negative control Kaybonnet rice containing three copy equivalents of pSYN12424 plasmid DNA (lane 3), were subjected to restriction endonuclease digestion with *Asc*I plus *Xma*I followed by agarose gel electrophoresis and transfer onto nylon membrane. Blots were hybridized with the mixture of DIG- labelled backbone probes 1–4 (panel A) or backbone probe 5 (panel B). Following washing, hybridized probes and DIG-labelled molecular weight markers VII (lane 1) were visualized using a chemiluminescent detection system followed by electronic image capture. Lane 2 was blank on all gels (adapted from GR2E-FFP submitted study reports)

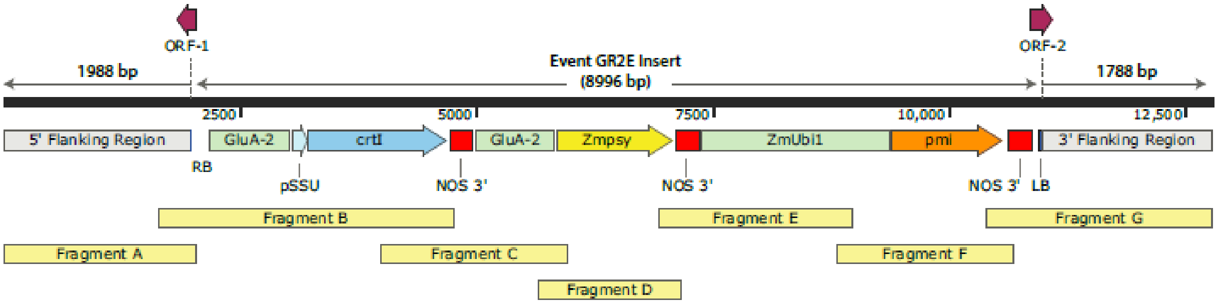

Supplementary Fig. 4 Schematic diagram of the GR2E T-DNA insert indicating the *crtI, Zmpsy1*, and *pmi* genes along with their respective regulatory elements. Approximate locations of the seven overlapping polymerase chain reaction (PCR) fragments subjected to nucleotide sequencing are indicated as A through G. The location of two putative ORFs spanning the 5' and 3' junctions of the T-DNA insert and the rice genomic DNA are indicated as ORF-1 and ORF-2, respectively (adapted from GR2E-FFP submitted study reports)

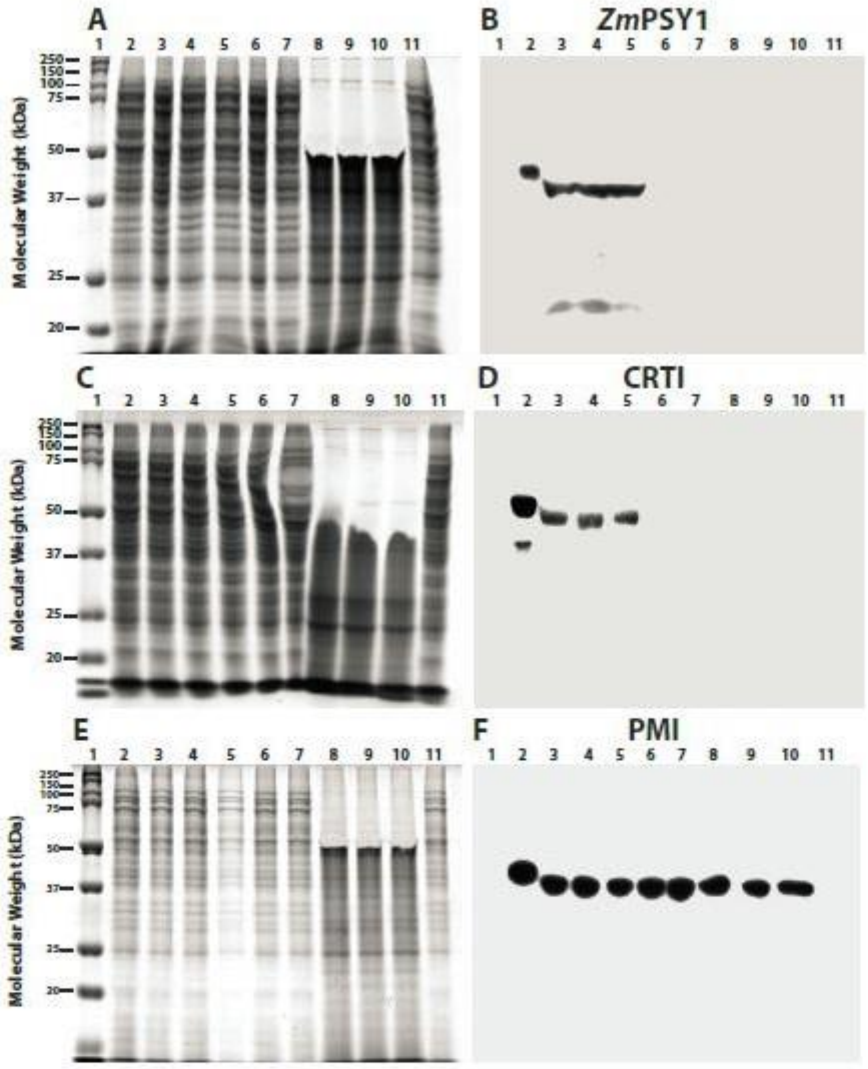

Supplementary Fig. 5 Total protein extracts were prepared from dough (BBCH 75; lane 3), milk (BBCH 85; lane 4), and mature (BBCH 90; lane 5) grain, bran (lane 6), hulls (lane 7), leaf (lane 8), stem (lane 9), and root tissue (lane 10) obtained from Kaybonnet rice containing event GR2E. Similar extracts were prepared from control non-transgenic Kaybonnet dough-stage grain (lane 11). For each analysis, the positive control sample (lane 2) consisted of control Kaybonnet extract spiked with 2.5 ng of purified ZmPSY1 protein (Lot No. M20452-05; panels A and B), 25 ng of purified CRTI protein (Lot No. M20454-02; panels C and D), or 6.25 ng purified PMI protein (Lot No. 21038G; panels E and F). Samples (ca. 40 µg total protein, panels A–D, or 7 µg total protein, panels E and F) were subjected to sodium dodecylsulfate (SDS)-polyacrylamide gel electrophoresis (PAGE). Gels were either stained for total protein with colloidal blue G250 (panels A, C, and E) or subjected to western immunoblot analysis (panels B, D, and F). For ZmPSY1 (panel B) and CRTI (panel D), the blots were labelled with monoclonal anti-ZmPSY1 (ELX1048; 2 µg/ml) and monoclonal anti-CRTI (ELX1043; 2 µg/ml), respectively, followed by alkaline phosphatase (AP)- conjugated goat anti-mouse IgG (1:10000). For PMI (panel F), the blot was labelled with horseradish peroxidase (HRP)-conjugated rabbit anti-PMI (1:100). For all blots, detection of bound antibody-conjugate was via chemiluminescent substrate development. Molecular weight standards are shown in lane 1 (panels A, C, and E) (adapted from GR2E-FFP submitted study reports).

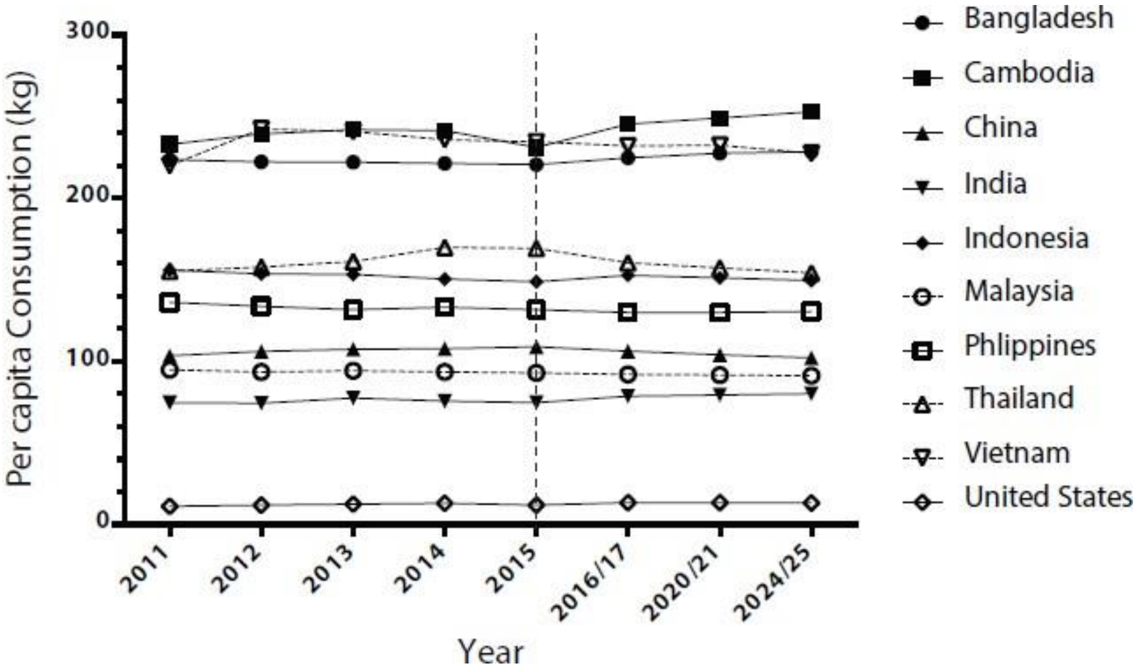

Supplementary Fig. 6 Per capita rice consumption in the highest-consuming countries in Asia compared with the United States. Values from 2011–2015 were derived using the USDA Production, Supply, and Distribution online database (USDA-FAS, 2016) and the FAOSTAT population database, while values after 2015 are projections taken from Wailes and Chavez (2015) (adapted from GR2E-FFP submitted study reports).

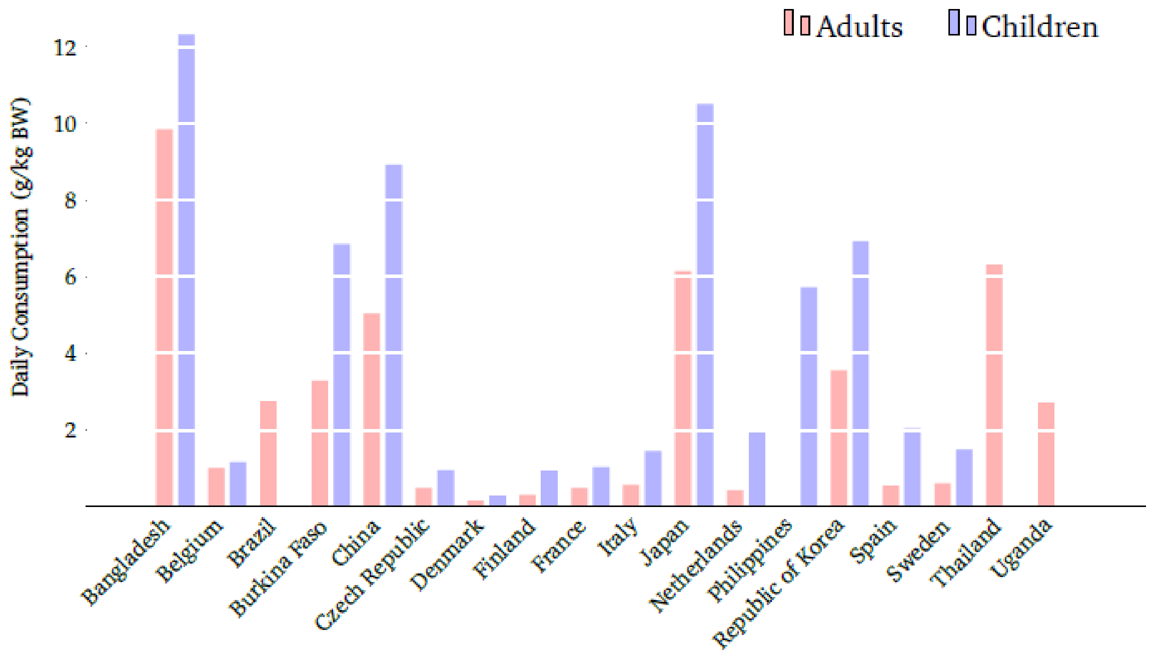

Supplementary Fig. 7 Daily individual rice consumption rates for adults and children in selected countries using data from the FAO/WHO Chronic Individual Food Consumption Database (adapted from GR2E-FFP submitted study reports)

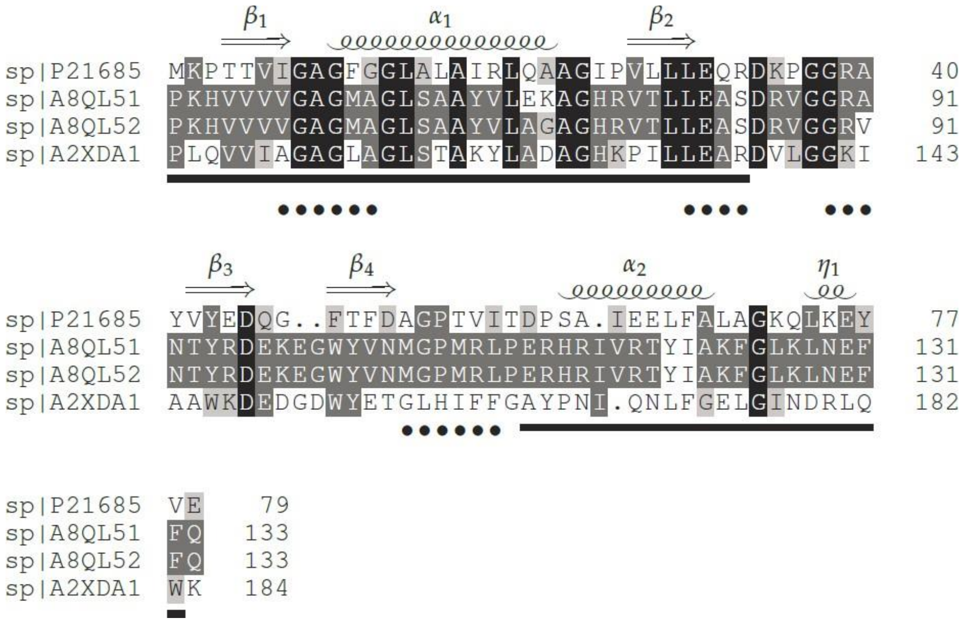

Supplementary Fig. 8 Alignment of the N-terminal region of the *P. ananatis* CRTI protein (spjP21685, residues 1–79) with the corresponding regions of the rice (*O. sativa*) phytoene desaturase (OsPDS, spjA2XDA1) and the two L-amino acid oxidase enzymes identified from the toxin database search. These were from *Bungarus multicinctus* (OXLA_BUNMU, spjA8QL51) and *B. fasciatus* (OXLA_BUNFA, spjA8QL52). The secondary structure elements of CRTI have been indicated above the alignment and the shaded bar underneath the alignment indicates the FAD-binding domains within the N-terminal region, with bullets indicating putative FAD binding residues, as described in Schaub et al. (2012). This figure was generated with TEXshade (Beitz, 2000) (adapted from GR2E-FFP submitted study reports).

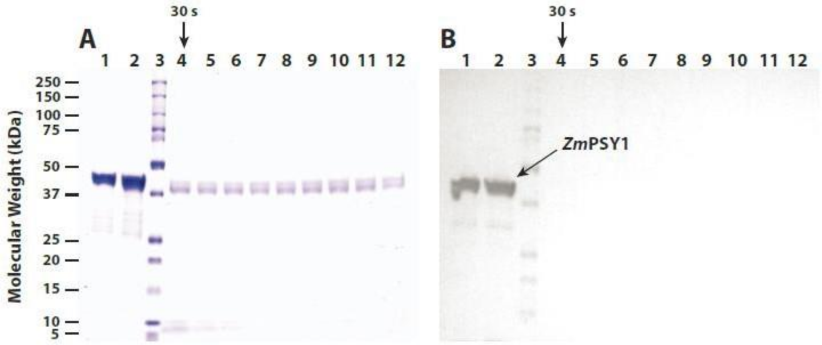

Supplementary Fig. 9 Panels A and B: Samples of *Zm*PSY1 protein purified from recombinant *E. coli* (Lot No. M20452-05) were incubated in the presence of SGF pH 1.2 containing pepsin for 0 min (lane 2) and 0.5, 1, 2, 5, 10, 20, 30 or 60 min at 37°C (lanes 4–11) and then analyzed by SDS-PAGE. Gels were either stained for protein with colloidal blue G250 (panel A) or subjected to western immunoblot analysis (panel B) using rabbit anti-*Zm*PSY1 immunoglobulin and horseradish peroxidase-conjugated goat anti-rabbit IgG followed by precipitating substrate development. Control samples included *Zm*PSY1 protein diluted in gastric control fluid without pepsin (lane 1) and SGF solution containing pepsin (lane 12). Molecular weight standards are shown in lane 3. The apparent molecular weight of *Zm*PSY1 protein corresponds to ca. 42 kDa (adapted from GR2E-FFP submitted study reports).

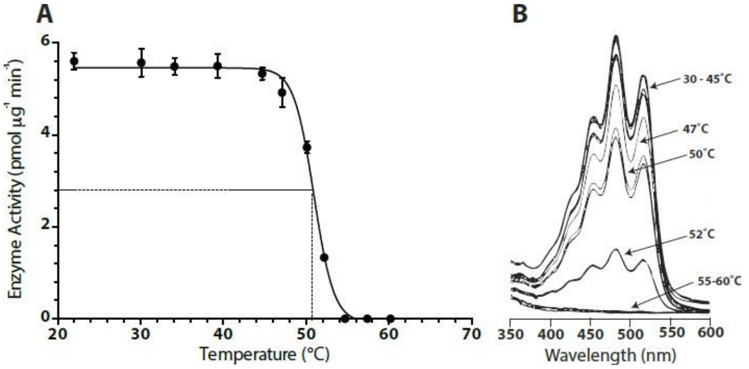

Supplementary Fig. 10 Individual samples of CRTI protein purified from recombinant E. coli (Lot No. M20454-02) were heated for 15 minutes at a designated temperature ranging from 30–60°C. Following this treatment, enzymatic conversion of 15-cis-phytoene to all-trans-lycopene was measured with a spectrophotometric assay. **Panel A** shows enzymatic activity (pmol $\mu g^{-1} min^{-1}$) versus pre-incubation temperature, where the values are means of two technical replicates and error bars represent the standard deviation around the mean value. **Panel B** illustrates the UV-visible spectral scans (350–600 nm) of the chloroform:methanol extracts of CRTI activity assays following different pre-incubation temperature regimes. The maximum absorbance at 483 nm was used to calculate the amount of all-trans-lycopene product (adapted from GR2E-FFP submitted study reports).

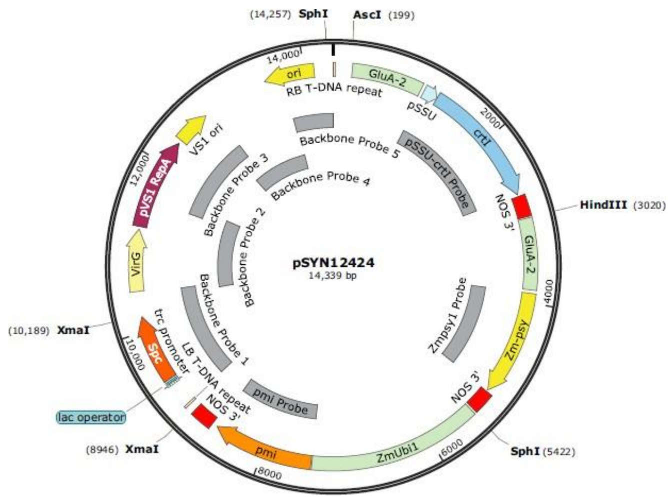

Supplementary Fig. 11 Schematic map of plasmid pSYN12424 showing the location of the DNA probes used for Southern hybridization characterization of GR2E rice (adapted from GR2E-FFP submitted study reports).

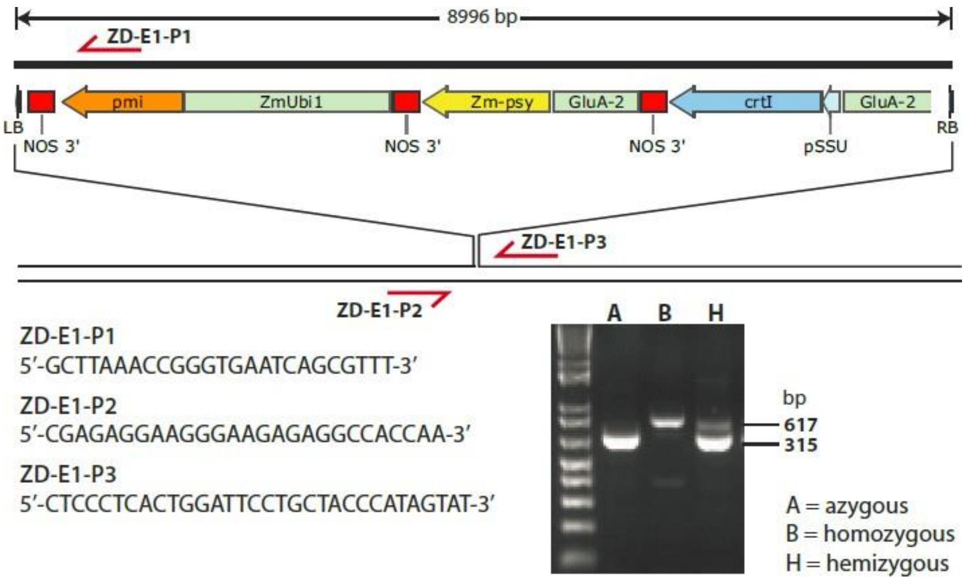

Supplementary Fig. 12 Genomic DNA isolated from individual plants was analyzed by multiplex PCR amplification employing three primers. Two primers, ZD-E1-P2 and ZD-E1-P3, were complementary to sequences in the left border (LB) and right border (RB) flanking host genomic regions, respectively, and primer ZD-E1-P1 was complementary to pmi gene sequences within the inserted T-DNA. Null segregants lacking the pSYN12424 T-DNA resulted in amplification of a single 315 bp fragment. Homozygous GR2E plants resulted in amplification of a single 617 bp fragment, and hemizygous plants resulted in the amplification of two fragments (315 bp and 617 bp) (adapted from GR2E-FFP submitted study reports).

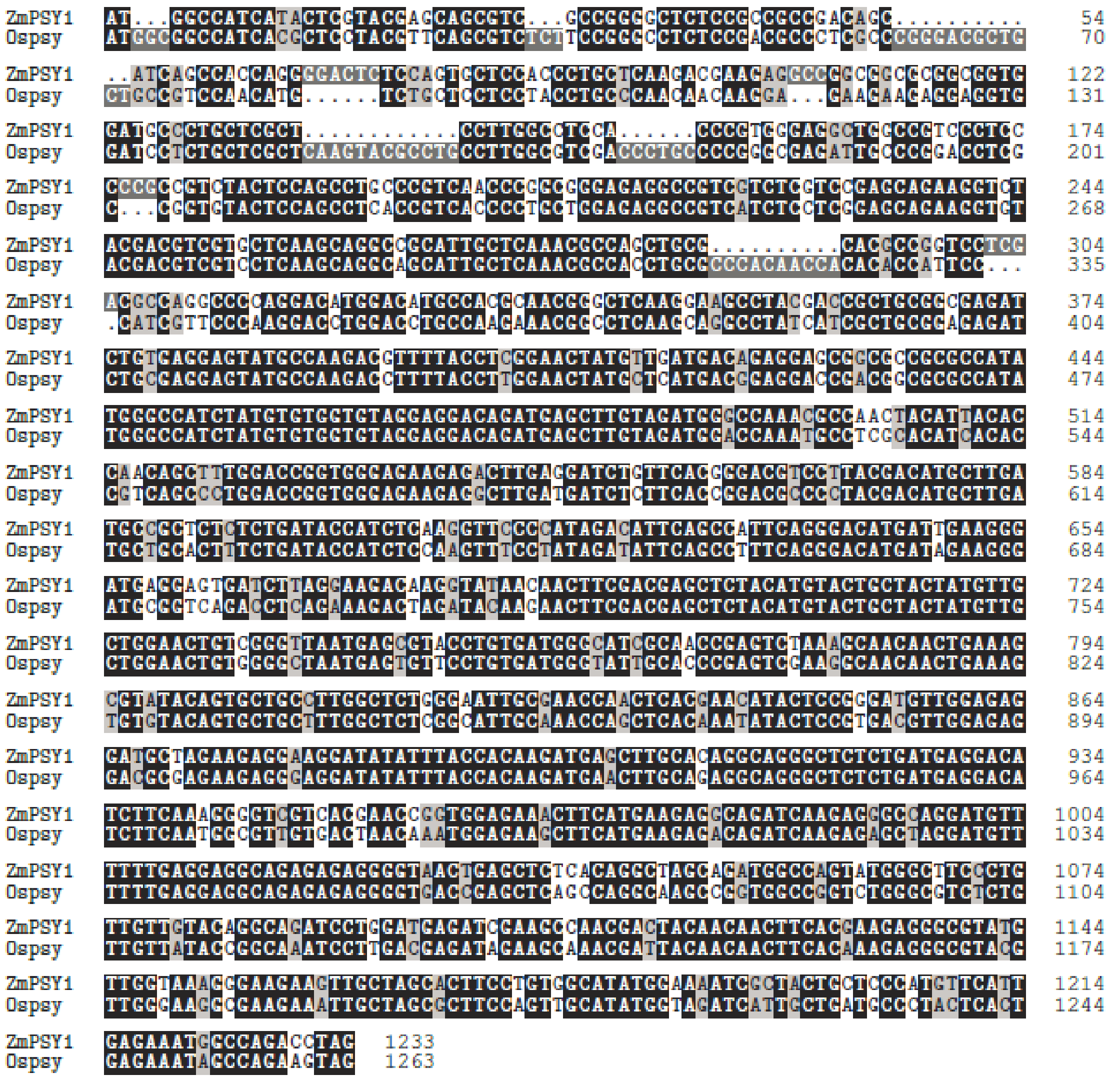

Supplementary Fig. 13 Alignment of the nucleotide sequences of the *Zea mays psy1* (*Zmpsy1*; GI:1098664) and *Oryza sativa psy* (*Ospsy*; GI:47678186) genes. These two genes share 86.9 percent similar residues and 82.8 percent identical residues. This figure was generated with TEXshade (Beitz, 2000)

Supplementary Table 1 Location and sizes of DNA probes used for Southern hybridization

Probe	Primers (5' to 3')	Position on pSYN12424 (bp to bp)	Length (bp)
Zmpsy1	ATGGCCATCATACTCGTACGA<br>CTAGGTCTGGCCATTTCTCAA	3893-3913<br>5105-5125	1233
SSU-crtI	ATGGCTTCTATGATATCCTCTTCC<br>TCAAATCAGATCCTCCAGCA	1087-1110<br>2717-2736	1650
pmi	ATGCAAAAACTCATTAACTCAGTGC<br>TTACAGCTTGTTGTAAACACGC	7429-7453<br>8583-8604	1176
Backbone 1	CCACCAGCCAGCCAACAG<br>CTTHTTCACAGCGCTTGAGA	9028-9045<br>10418-10437	1410
Backbone 2	GCCGCAATTCTGACGAACTG<br>CGGTTCATGGATTCGGTTAG	10355-10374<br>11664-11683	1329
Backbone 3	GTGATCCGCTACGAGCTTCC<br>CTCTCATCAACCGTGGCTCC	11559-11578<br>12821-12840	1282
Backbone 4	CTACGGCCAGGCAATCTACC<br>TGGAGCGAACGACCTACACC	12681-12700<br>13751-13770	1090
Backbone 5	ATCTCAGTTCGGTGTAGGTC<br>CTAAGAGAAAAGAGCGTTTATTAG	13741-13760<br>14316-14339	599

(adapted from GR2E-FFP submitted study reports)

Supplementary Table 2 Correlation of predicted and observed fragment sizes bases on a single site of insertion of the pSYN12424 T-DNA within GR2E rice

		Event GR2E Genomic DNA Fragment Size (bp)		Plasmid pSYN12424 Fragment Size (bp)	
Probe	Enzyme	Predicted	Observed[a]	Predicted	Observed
Zmpsy1	*Asc*I + *Xma*I	8747	8747	-	-
	*Sph*I	>5422	~6900<br>~5600[b]	5504	5504
	*Hin*dIII	>6010	~7900<br>~4900[b]	-	-
SSU-crtI	*Asc*I + *Xma*I	8747	8747	-	-
	*Sph*I	>5422	~6900	5504	5504
	*Hin*dIII	>3020	~7200	-	-
pmi	*Asc*I + *Xma*I	8747	8747	-	-
	*Sph*I	>3608	~5500	8835	8835
	*Hin*dIII	>6010	~7900	-	-

[a] Observed fragment sizes were approximated from the digoxigenin (DIG)-labelled DNA molecular size marker VII fragments on the Southern blots. Due to the inability to determine exact sizes on the blot, all values are approximations to the nearest 200 bp. The exception to this was when the observed fragment had equal migration as the control plasmid hybridization fragment, in which case the predicted size was assigned (e.g., 5504 bp in the case of *Sph*I digestions probed with DIG-labelled *Zmpsy1* or *SSU-crtI*).

[b] These fragments were due to hybridization with sequences from the endogenous rice *psy* gene, which is ca. 83 percent identical to the *Zmpsy1* gene . (Supplementary Fig.13,) (adapted from GR2E-FFP submitted study reports).

Supplementary Table 3 Primers used for nucleotide sequencing of the inserted DNA within GR2E rice

PCR Fragment	Primer	Primer Sequences (5'-3')	Target Sequence Location (bp to bp)†	Amplified Fragment Size (bp)
A	GR2E_F1<br>GR2E_R1	TCTGGAGGATCTACTGCTGC<br>CGCTCATGATCAGATTGTCG	1-20<br>2022-2041	2041
B	GR2E_F2<br>GR2E_R2	CCCAGTGAGTAGTATTTTCAGTC<br>GT<br>AAGACCGGCAACAGGATTC	1642-1666<br>4780-4778	3137
C	GR2E_F3<br>GR2E_R3	TAGAGGACGGTCGCAGGTTC<br>GGCCTCTTCGTCTTGAGCAG	3998-4017<br>5945-5964	1967
D	GR2E_F4<br>GR2E_R4	TCCACCTTTCGTGTACCACA<br>AAGACCGGCAACAGGATTC	5662-5681<br>7149-7168	1506
E	GR2E_F5<br>GR2E_R5	GGATGAGATCGAAGCCAACG<br>CCACATCATCACAACCAAGC	6952-6971<br>8820-8969	2018
F	GR2E_F6<br>GR2E_R6	GGATCTGTATGTGTGTGCCA<br>AAGACCGGCAACAGGATTC	8820-8839<br>10668-10686	1868
G	GR2E_F7<br>GR2E_R7	ACCACCATTAGCCAGCAGAG<br>AGGAAGAATGCATCTGGAGC	10390-10409<br>12753-12772	2383

† Numbering is relative to the sequence of the insert and flanking regions from event GR2E rice. Nucleotides corresponding to the 5' flanking sequence (1-1988), T-DNA insert (1989-10984), and 3' flanking sequence (10985-12772). (adapted from GR2E-FFP submitted study reports)

Supplementary Table 4 Thermocycling parameters

Fragment	Denaturation	Annealing	Extension	Cycles
A,B,G	3 min; 95ºC<br>20 sec; 98ºC	15 sec; 55ºC	2 min; 72ºC<br>7 min; 72ºC	1<br>35<br>1†
C	3 min; 95ºC<br>20 sec; 98ºC	15 sec; 55ºC	2 min; 72ºC<br>7 min; 72ºC	1<br>35<br>1
D	3 min; 95ºC<br>20 sec; 98ºC	15 sec; 55ºC	90 sec; 72ºC<br>7 min; 72ºC	1<br>35<br>1
E	3 min; 95ºC<br>20 sec; 98ºC	15 sec; 65ºC	2 min; 72ºC<br>7 min; 72ºC	1<br>35<br>1
F	3 min; 95ºC<br>20 sec; 98ºC	15 sec; 60ºC	2 min; 72ºC<br>7 min; 72ºC	1<br>35<br>1

† Following completion of the final extension incubation, samples were held at 4ºC. (Adapted from GR2E-FFP submitted study reports)

Supplementary Table 5 Mendelian Inheritance of the pSYN12424 T-DNA insert within multiple generations of GR2E rice

		Expected		Observed			
Generation[a]	Total	Present	Absent	Present	Absent	Chi Square[c]	p-Value
BC4F2*1 (3:1)	64	48	16	44	20	1.333	0.2482
BC4F2*2 (3:1)	51	38.25	12.75	38	13	0.007	0.9356
BC4F2*3(3:1)	63	47.25	15.75	43	20	1.529	0.2162
BC5F1*1 (1:1)	59	29.5	29.5	32	27	0.424	0.5151
BC5F1*2 (1:1)	49	24.5	24.5	25	24	0.020	0.8864
BC5F1*3 (1:1)	127	63.5	63.5	68	59	0.638	0.4245
BC5F2*1 (3:1)	100	75	25	74	26	0.053	0.8174
BC5F2*2(3:1)	99	74.25	24.75	72	27	0.273	0.6015
BC5F2*3(3:1)	100	75	25	70	30	1.333	0.2482

[a] Generation identified with the superscript "*1", "*2", and "*3" designation represented different GR2E breeding lines where the recurring non-transgenic parent was PSB Rc82, BRRI *dhan* 29, and IR64, respectively.

[b]Multiplex PCR-based zygosity testing was performed on each plant from each generation.

[c]The analysis tested the hypothesis that the introduced DNA was segregating in a Mendelian fashion. The critical value to reject the hypothesis at the five percent level is 3.84. Since the Chi-square (χ^2) value was less than 3.84 within each generation, the observed differences were not statistically significant(adapted from GR2E-FFP submitted study reports).

Supplementary Table 6 Location and identity of the ORFs spanning the 5' and 3' T-DNA–genomic DNA junctions in GR2E rice

ID	Nucleotide Location	Strand	Length (amino acids)	Deduced Amino Acid Sequence	No. of Allergen Hits	No. of FASTA Hits[a]
ORF-1	1830-2036	-	68 (7.9 kDa)	MIRLSFPAFSLNYQCL EKKRRFISLPVHLHQF CNCNSGCVYVSIHIHT HTLNFQTILNRLSILSP VLP	None	None
ORF-2	10870-11109	+	79 (8.8 kDa)	MLLDLLALQEIYRCPG GQHGRIRNVLLSCLSV NLFTPQFLGNGRCNQS MTTRLESKQAQILDQF IGGLSSLPLAFLLLQ	None	None

[a]Sequences were queried against a protein toxin database using FASTA with an *E*-value cutoff of 1 x 10^{-5} (adapted from GR2E-FFP submitted study reports).

Supplementary Table 7 Concentrations of *Zm*PSY1, CRTI, and PMI in samples of grain and straw collected from GR2E rice grown at four locations in the Philippines during two growing seasons in 2015-16

	Concentration (ng/g FWT)†					
	*Zm*PSY1		CRTI		PMI	
Samples	2015	2016	2015	2016	2015	2016
Grain (milk stage)	156.6 (126.4-180.8)	161.7 (138.5-184.6)	43.6 (23.3-52.2)	36.6 (31.4-40.0)	1548.1 (1097-1798)	1828.4 (1352-2083)
Grain (dough stage)	338.6 (326.3-358.9)	328.9 (308.2-356.4)	61.8 55.3-67.6)	60.3 (53.8-68.0)	1897.9 (1565-2197)	2130.7 (1912-2397)
Grain (mature stage)	226.6 (196.7-244.8)	220.2 (195.7-239.6)	26.1 (23.0-29.7)	22.8 (16.6-27.4)	1369.0 (915-1891)	1195.0 (765-1780)
Straw (mature stage)	<LOQ[a]	<LOQ	<LOQ	<LOQ	466.7 (320-617.5)	498.0 (339-795.5)

† Values represent the mean across four locations of three replicate samples collected from each location in the Philippines where event GR2Erice was grown during the rainy season in 2015 and again during the dry season in 2016 (n=12 for each season). The lowest and highest individual means location values for each year are shown in parentheses. Concentrations are uncorrected for extraction effici ency and expressed in ng protein per gram fresh weight tissue (FWT).

[a]LOQ = Limit of quantification, which for the *Zm*PSY ELISA with straw (stem) samples was 66.4 ng/g FWT and for CRTI with straw samples was 6.2 ng/g FWT. (adapted from GR2E-FFP submitted study reports)

Supplementary Table 8 Historic and projected per capita rice consumption in Asia and in the US

Country	Annual per capita Utilization (kg)[a]					Projected per capita Utilization (kg)[b]		
	2011	2012	2013	2014	2015	2016/17	2020/21	2024/25
Bangladesh	223.6	222.2	222.1	221.3	220.5	224.8	227.6	228.5
Cambodia	233.0	239.3	242.1	241.4	231.1	245.4	249.1	252.9
China	103.5	106.2	107.4	107.8	109.0	106.3	104.1	102.1
India	74.8	74.4	77.5	76.7	74.7	78.6	79.4	80.1
indonesia	156.0	153.7	153.2	150.5	148.7	152.7	151.2	149.5
Malaysia	94.8	93.5	94.2	93.6	93.0	92.1	91.7	91.3
Philippines	136.1	133.8	131.7	133.1	131.6	130.0	130.0	130.5
Thailand	155.4	157.8	161.2	169.8	169.2	160.4	157.2	154.2
Vietnam	220.0	242.4	240.8	235.9	234.4	232.0	232.5	227.6
United States	11.2	12.0	12.5	13.0	12.0	13.5	13.5	13.3

[a]Consumption estimates were derived using data from the USDA PSD (Production, Supply and Distribution) online database and the FAOSTAT population database.

[b]Projection excerpted from Table 68 in Wailes and Chavez (2015). (adapted from GR2E-FFP submitted study reports)

Supplementary Table 9 FAO/WHO CIFOCOss per capita daily rice consumption data for selected countries in Asia

Country	Population Subgroup	Number of Consumers Sampled	Mean Daily Consumption (g/kg body weight)[a]
Bangladesh	Adult women	474	9.84
	Children	545	12.32
China	General population	50011	5.04
	Children	2,233	8.92
Japan	General population	2711	6.15
	Children	71	10.5
Philippines	Children	1114	5.72
Republic of Korea	General population	9225	3.56
	Children	648	6.92
Thailand	General population	15695	6.31

[a]Individual consumption data for rice (excluding wild rice: GC 0649) were obtained from the FAO/WHO Chronic Individual Food Consumption Database summary statistics (CIFOCOss) accessed on 16 May 2016. Available at http://www.who.int/foodsafety/databases/en/. (adapted from GR2E-FFP submitted study reports)

Supplementary Method 1 Nucleotide Sequence Analysis of GR2E Rice

The nucleotide sequence of the plasmid pSYN12424 T-DNA, together with preliminary sequence information from the 5' and 3' flanking genomic DNA (Syngenta), was used to design seven sets of oligonucleotide primers that were used to amplify the insert and flanking regions from GR2E rice genomic DNA as seven individual overlapping fragments (Supplementary Table 3). PCR amplifications were carried out using the KAPA HiFi HotStart PCR Kit (Kapa Biosystems, Wilmington, MA, USA) and a G-Storm GS1 thermocycler using the parameters in Table S4.

The PCR amplification fragments were cloned into pCR4-TOPO vector via blunt-end ligation and transformed into *E. coli* strain DH5. Three colonies from each transformation were randomly selected and separately transferred into liquid culture and grown overnight at 37°C with shaking. Plasmid DNA prepared from each culture was submitted to Macrogen Incorporated (Seoul, Korea) for dye-terminator nucleotide sequencing using the ABI3730XL analyzer with ABI BigDye 3.1 terminator chemistry. Sequence analysis was performed using the Phred, Phrap, and Consed packages (University of Washington) to an error rate of less than 1 in 10,000 bases (Ewing and Green, 1998). Nucleotide sequences from each individual clone were aligned using AlignX™ (Vector NTI Advance™ v. 11.0) to obtain the final consensus sequence for each PCR amplification fragment.

Supplementary Method 2 Sequence Similarity Searches to Known Allergens using ORF-1 and ORF-2 Sequences as Queries

The deduced amino sequences of the two junction-spanning open reading frames (ORFs) identified in GR2E rice were used as query sequences to search for significant sequence similarities with 2129 known and putative allergen and celiac protein sequences residing in the FARRP19 dataset at the University of Nebraska. Search routines included a search for full-length alignments using FASTA to identify any significant sequence alignments (E-value threshold $1X10^{-5}$) that would be indicative of possible cross-reactivity, as well as the sliding 80-mer search to look for >35 percent sequence identity over all possible 80 amino acid segments and a search for exact matches of 8 contiguous amino acids.

Supplementary Reference

Beitz, E. TEXshade: shading and labeling of multiple sequence alignments using LATEX2 epsilon. *Bioinformatics (Oxford, England)*, 16(2):135–139 (2000).
